# Supplementary material for: In Search of a Universal Method: A Comparative Survey of Bottom-Up Proteomics Sample Preparation Methods
Source: J Proteome Res. 2022 Aug 25;21(10):2397–411. doi: 10.1021/acs.jproteome.2c00265 (PMC9552232; doi:10.1021/acs.jproteome.2c00265)
Supplement: Supplementary file 1 — pr2c00265_si_001.pdf [file pr2c00265_si_001.pdf]

In search of the universal method: a comparative survey of bottom-up proteomics sample preparation methods

Gina Varnavides[1], Moritz Madern[1,2], Dorothea Anrather[1], Natascha Hartl[1], Wolfgang Reiter[1,2], Markus Hartl[1,2]*

[1]Mass Spectrometry Facility, Max Perutz Labs, Vienna BioCenter, Vienna, Austria
[2]Department of Biochemistry and Cell Biology, Max Perutz Labs, University of Vienna, Vienna BioCenter, Vienna, Austria

* Corresponding author information: markus.hartl@univie.ac.at

Table of Content

Supplemental Figure 1	page S-3
Supplemental Figure 2	page S-5
Supplemental Figure 3	page S-6
Supplemental Figure 4	page S-7
Supplemental Table Legends	page S-9
References	page S-10

Supplemental Figures

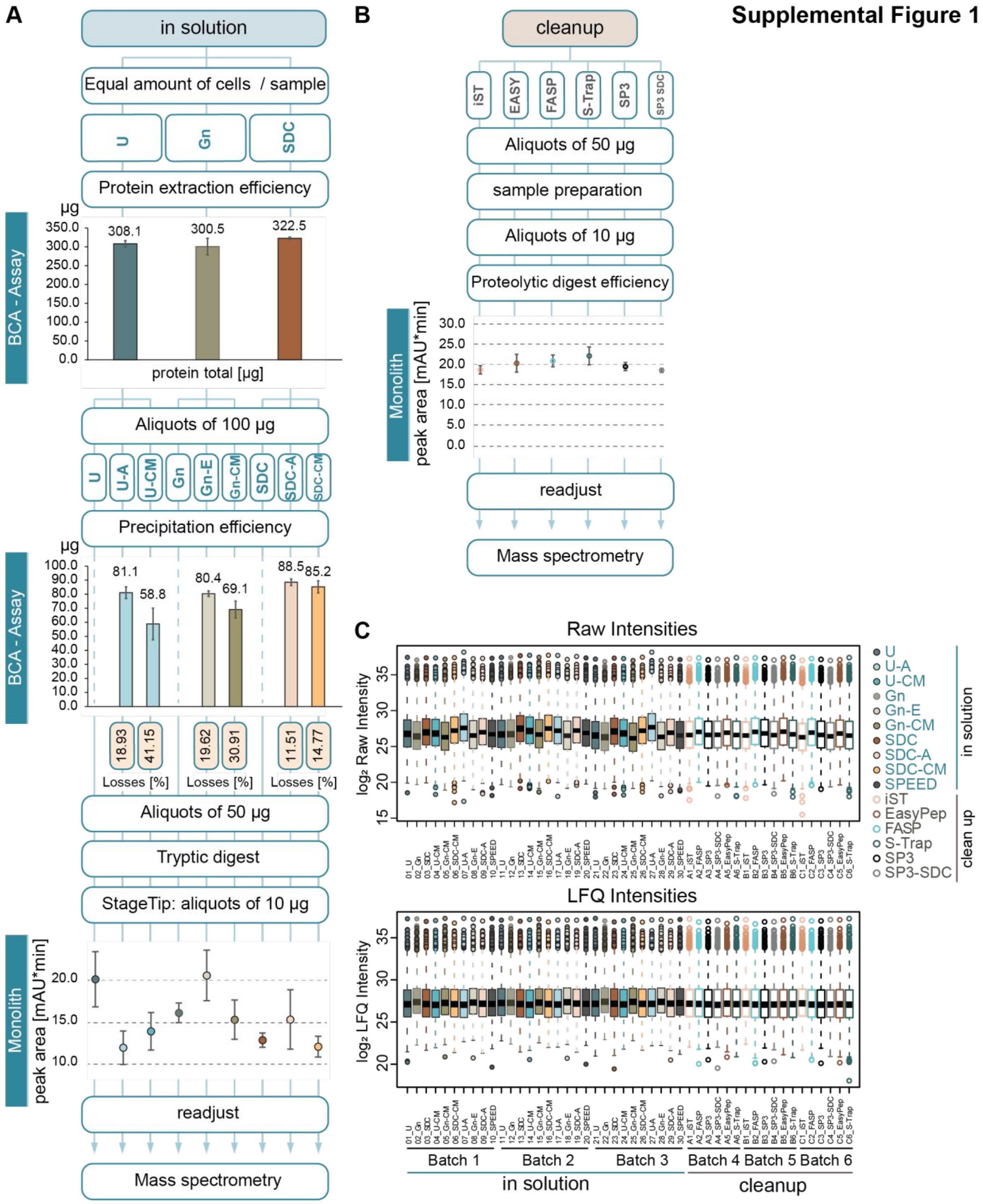

Supplemental Figure 1 - Selected quality control steps from our experimental approach. Supplement to Figure 1. **(A)** Scheme illustrating experimental workflow for ISD samples, including results from quality control testing. HeLa proteomes were extracted using urea (U)-, guanidine hydrochloride (G)-, or sodium deoxycholate (SDC)-buffered systems. Upper bar plot indicates extraction efficacy determined using the BCA™ Protein assay kit (Thermo Scientific). Lysates were either directly submitted to tryptic digestion or precipitated using acetone (A), ethanol (E) or chloroform/methanol (CM). Central bar plot: Precipitation efficacy was determined using the BCA™ Protein assay kit (Thermo Scientific). Tryptic digests were desalted using C18 stage tips and efficacy of proteolysis was determined by quantifications of UV chromatogram peak areas. Samples were adjusted to ensure equal loading for MS measurements. **(B)** Scheme illustrating experimental workflow for cleanup samples, including quantifications of UV chromatogram peak areas. **(C)** Box plots showing distributions of non-normalized log_2 protein group intensities (top) as well as normalized log_2 LFQ intensities (bottom) for each sample (y-axes). LC-MS batch numbers are indicated below samples (x-axis).

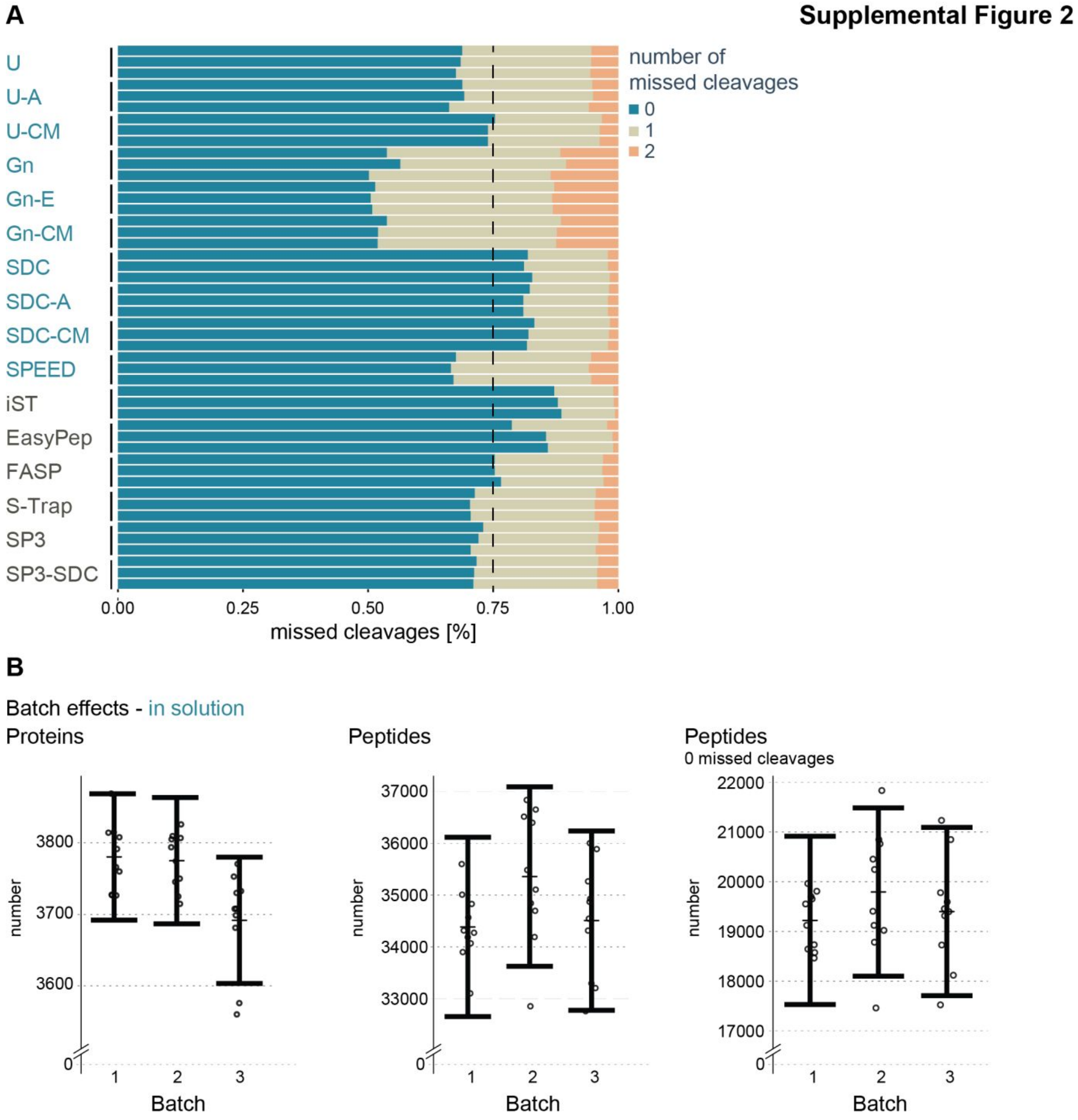

Supplemental Figure 2 – Missed cleavages and batch effects. Supplement to Figure 2. (**A**) Bar diagram indicating number of single, double, triple missed cleavage peptides in percent. Diagram has been adapted from the PTXQC [1] report. (**B**) Partial Residual Plots depicting batch effects of MS measurements on the number of identified proteins (left), identified peptides (middle) and peptides with no missed cleavages (right). Data points represent the number of IDs. Error bars correspond to a 95% confidence interval (CI). Black lines indicate the average number of IDs.

Supplemental Figure 3

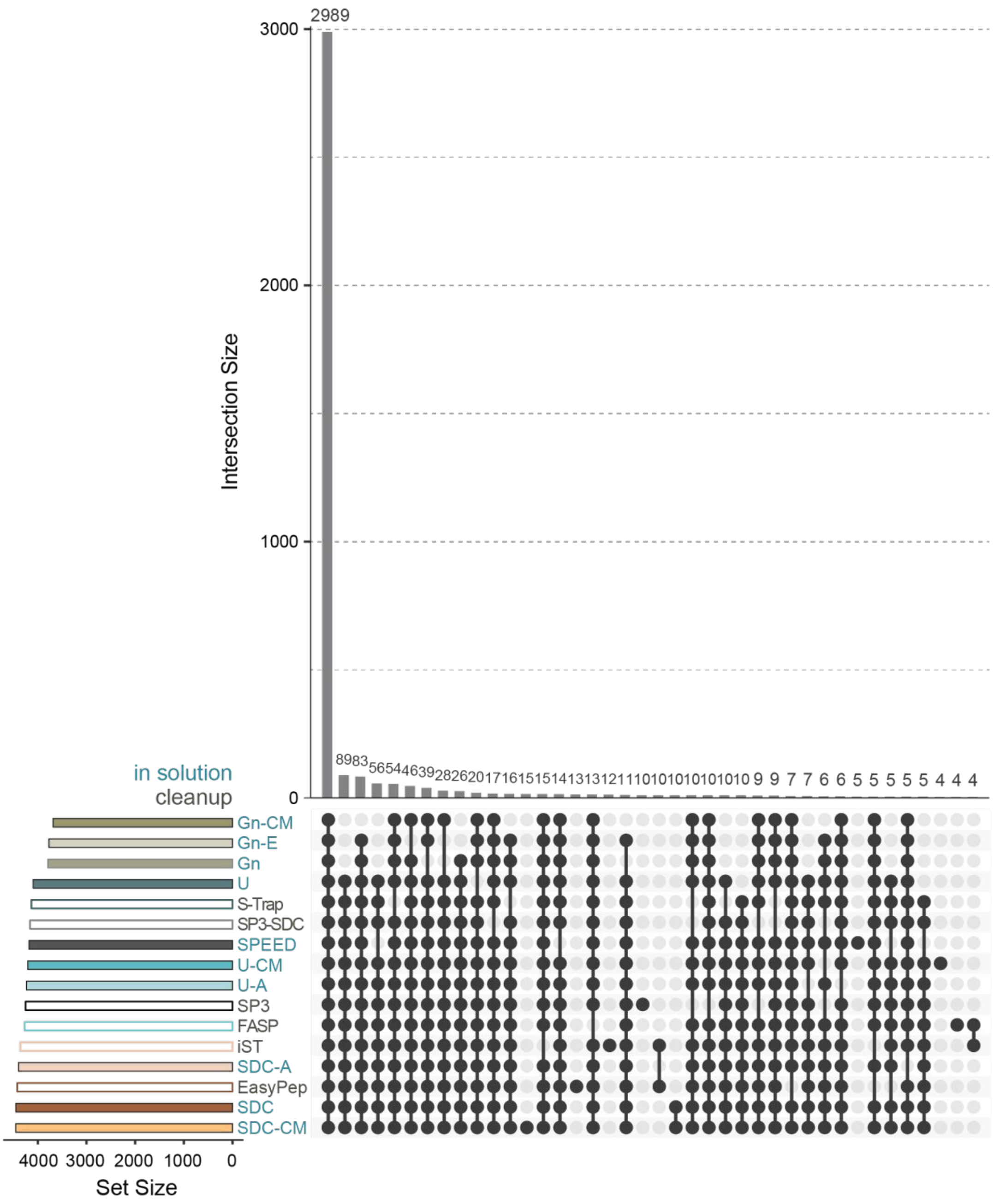

Supplemental Figure 3 - Overlap of identified proteins between all methods. Supplement to Figure 4. UpSet plot visualizing intersections of protein IDs extracted by individual sample preparation methods (sets) in a matrix layout. (Top) The x-axis shows intersections of set combinations through gray bars which are labeled with their respective intersection size. (Bottom) The overall set-size of a sample preparation method is listed on the bottom left. Next to each method (row), a black dot represents the inclusion of the respective set in an intersection (column). Sets that are not included in an intersection appear light gray.

Supplemental Figure 4

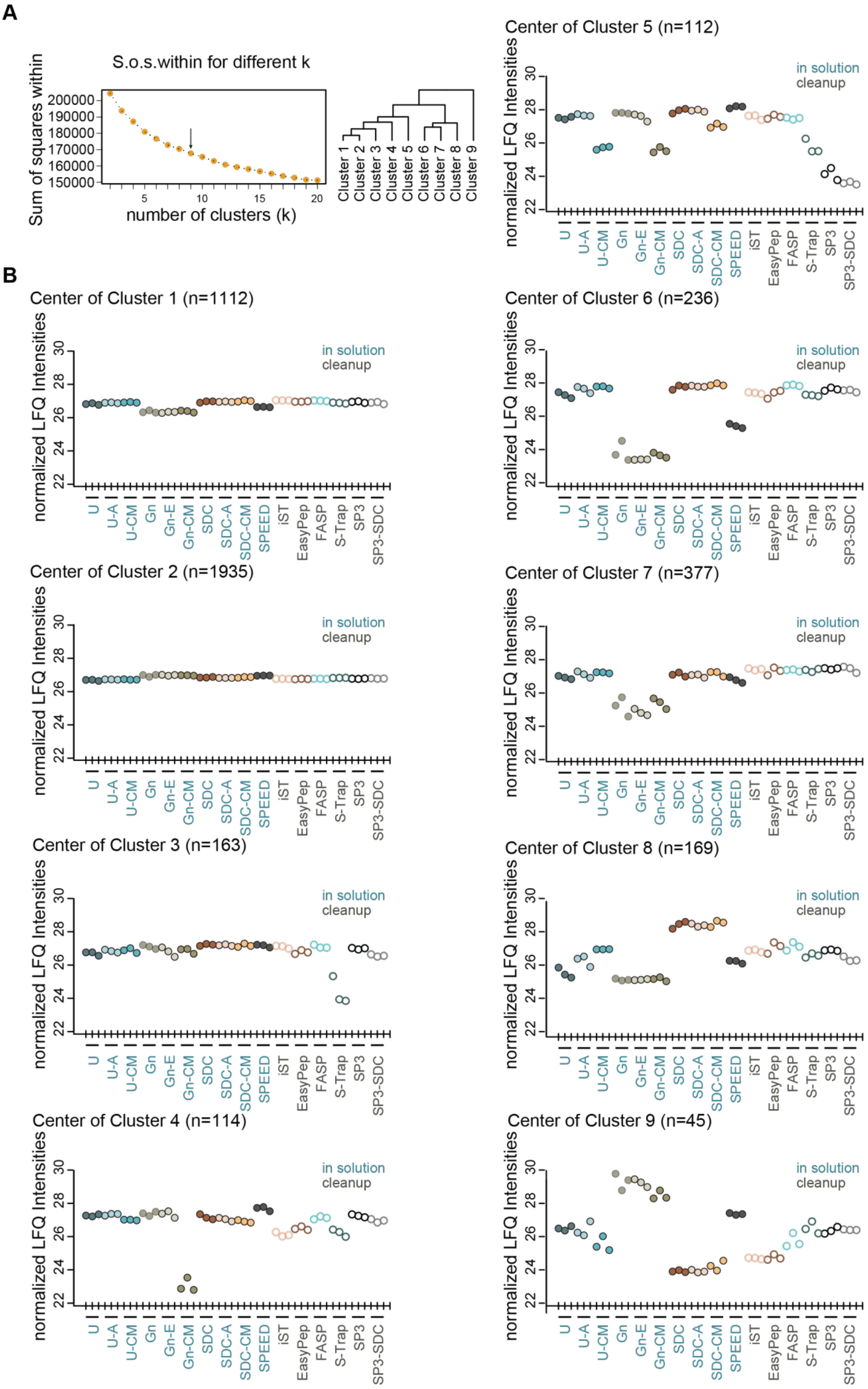

Supplemental Figure 4 – K-means cluster centers. Supplement to Figure 5. **(A)** Left: K-means plot: optimal number of clusters k was determined based on the total sum of squares within (SSW) for different k. Nine clusters were defined (s*ee material and methods*). Right: dendrogram of cluster centers as a result of an agglomerative clustering of k-means cluster centers with ultrametric euclidean distance. **(B)** Profiles of all k-means clusters. Dots represent method's normalized LFQ intensities.

Supplemental Table Legends

Supplemental Table 1 – Number of proteins, peptides and missed cleavages. Supplement to Figure 2. Table listing total number of identified (by MS/MS) proteins (sheet 1), peptides (sheet 2) and peptides with no missed cleavages (sheet 3).

Supplemental Table 2 – Open search results. Supplement to Figure 3. Results obtained from the open search with MSFragger analysis output table “global.modsummary.tsv”. Sum of PSMs of corresponding replicates of all samples are shown in numbers (left) and percent (right). Sheet 2: MSFragger output table “global.profile.tsv”.

Supplemental Table 3 – Enrichment analysis. Supplement to Figure 5. Full matrix depicting enrichment and significance of protein features of the exploratory k-Means cluster analysis shown in Figure 5D. Columns: k-means clusters 1 - 9. Rows: protein features in each k-means cluster. Color code indicates enrichment factor of protein features.

References

(1) Bielow, C.; Mastrobuoni, G.; Kempa, S. Proteomics Quality Control: Quality Control Software for MaxQuant Results. *J. Proteome Res.* **2016**, *15* (3), 777–787. https://doi.org/10.1021/acs.jproteome.5b00780.
