# Supplementary material for: In Search of a Universal Method: A Comparative Survey of Bottom-Up Proteomics Sample Preparation Methods
Source: J Proteome Res. 2022 Aug 25;21(10):2397–411. doi: 10.1021/acs.jproteome.2c00265 (PMC9552232; doi:10.1021/acs.jproteome.2c00265)
Supplement: Supplementary file 5 — pr2c00265_si_005.zip [file pr2c00265_si_005.zip › Supplemental_Material_Scripts/Cassiopeia_Output/Cassiopeia_LFQ_07052021.pdf]

LFQ data analysis of XYZ

May 7, 2021

Contents

1 Chosen Parameters 2

2 Quality Control and Initial Filtering 4
2.1 Sample Names . . . 4
2.2 Distribution of Protein Scores . . . 4
2.3 Initial Filtering . . . 5
2.4 Checking Normalization . . . 5
2.4.1 Based on Boxplots . . . 5
2.4.2 Based on Scatterplots . . . 6
2.5 Checking Contamination and Top Proteins . . . 7

3 Advanced Filtering 12
3.1 Based on Contaminants . . . 12
3.2 Based on Razor + Unique Peptides . . . 13
3.3 Based on Valid Values . . . 13
3.4 Renormalization after Advanced Filtering . . . 13

4 Visualization before Imputation 14
4.1 Remaining Missing Values . . . 14
4.2 Heatmap before Imputation . . . 14
4.3 PCA before Imputation . . . 15

5 Imputation of Missing Values 16

6 Visualization after Imputation 23
6.1 Heatmap after Imputation . . . 23
6.2 PCA after Imputation . . . 24

7 Statistical Pairwise Comparison of Groups 25
7.1 Overview . . . 25
7.2 Results . . . 25

8 Exploratory Cluster Analysis with k-Means 25
8.1 Optimal k . . . 25
8.2 The k Cluster Centers . . . 26

9 Matrix Export 31

1 Chosen Parameters

These are the parameters used for generating this report:

```
print(filename)

## [1] "proteinGroups_gina (2020_11_13 19_25_46 UTC).txt"

print(groups)

##  [1] "LYS-UREA"   "LYS-GH"     "LYS-SDC"    "CMP-UREA"   "CMP-GH"
##  [6] "CMP-SDC"    "ACP-UREA"   "EP-UREA"    "ACP-SDC"    "SPEED"
## [11] "LYS-UREA"   "LYS-GH"     "LYS-SDC"    "CMP-UREA"   "CMP-GH"
## [16] "CMP-SDC"    "ACP-UREA"   "EP-UREA"    "ACP-SDC"    "SPEED"
## [21] "LYS-UREA"   "LYS-GH"     "LYS-SDC"    "CMP-UREA"   "CMP-GH"
## [26] "CMP-SDC"    "ACP-UREA"   "EP-UREA"    "ACP-SDC"    "SPEED"
## [31] "iST"        "FASP"       "SPC"        "SPC_SDC"    "EASY"
## [36] "STRAP"      "iST"        "FASP"       "SPC"        "SPC_SDC"
## [41] "EASY"       "STRAP"      "iST"        "FASP"       "SPC"
## [46] "SPC_SDC"    "EASY"       "STRAP"      "LysUREA_11"

print(export_matrix)

## [1] TRUE

print(export_amica)

## [1] FALSE

print(remove_contaminants)

## [1] TRUE

print(razor_plus_unique_peptides_filter)

## [1] TRUE

print(min_number_razor_plus_unique_peptides)

## [1] 2

print(mode_valid_values_filter)

## [1] "in_at_least_one_group"

print(number_valid_values_filter)

## [1] 3

print(renormalization_median)

## [1] FALSE

print(renormalization_quantile)
```

```
## [1] FALSE
print(renormalization_loess)
## [1] FALSE
print(renormalization_to_proteins)
## NULL
print(renormalization_to_sample)
## NULL
print(mode_imputation)
## [1] "constant"
print(downshift)
## [1] 1.8
print(width)
## [1] 0.3
print(pairwise_comp)
## NULL
print(trend_limma)
## [1] TRUE
print(batch)
## NULL
print(proteins_of_special_interest)
## NULL
print(number_of_clusters)
## [1] 9
print(reorder_samples_for_k_means_clustering)
## [1] TRUE
print(infer_optimal_number_of_clusters)
## [1] TRUE
print(export_clusters)
## [1] TRUE
```

Based on the parameter called groups, it was assumed that every experimental condition had the following number of replicates:

```
## warning: number of replicates per group either not uniform or > 5
```

2 Quality Control and Initial Filtering

2.1 Sample Names

These are the samples that Cassiopeia will be analyzing:

```
##  [1] "01_LYS-UREA"    "02_LYS-GH"      "03_LYS-SDC"     "04_CMP-UREA"
##  [5] "05_CMP-GH"      "06_CMP-SDC"     "07_ACP-UREA"    "08_EP-GH"
##  [9] "09_ACP-SDC"     "10_SPEED"       "11_LYS-UREA"    "12_LYS-GH"
## [13] "13_LYS-SDC"     "14_CMP-UREA"    "15_CMP-GH"      "16_CMP-SDC"
## [17] "17_ACP-UREA"    "18_EP-GH"       "19_ACP-SDC"     "20_SPEED"
## [21] "21_LYS-UREA"    "22_LYS-GH"      "23_LYS-SDC"     "24_CMP-UREA"
## [25] "25_CMP-GH"      "26_CMP-SDC"     "27_ACP-UREA"    "28_EP-GH"
## [29] "29_ACP-SDC"     "30_SPEED"       "A1_iST1"        "A2_FASP"
## [33] "A3_SP3"         "A4_SP3_SDC"     "A5_EASY"        "A6_STRAP"
## [37] "B1_iST"         "B2_FASP"        "B3_SP3"         "B4_SP3_SDC"
## [41] "B5_EASY"        "B6_STRAP"       "C1_iST"         "C2_FASP"
## [45] "C3_SP3"         "C4_SP3_SDC"     "C5_EASY"        "C6_STRAP"
## [49] "ref_LysUREA_11"
```

```
## In total: 49 samples
```

BTW: Make sure that your group specifications (in section 1: groups) corresponds to the sample order as it is listed here!

2.2 Distribution of Protein Scores

The following plot shows the distribution of Protein Scores as density for both reverse and non-reverse hits:

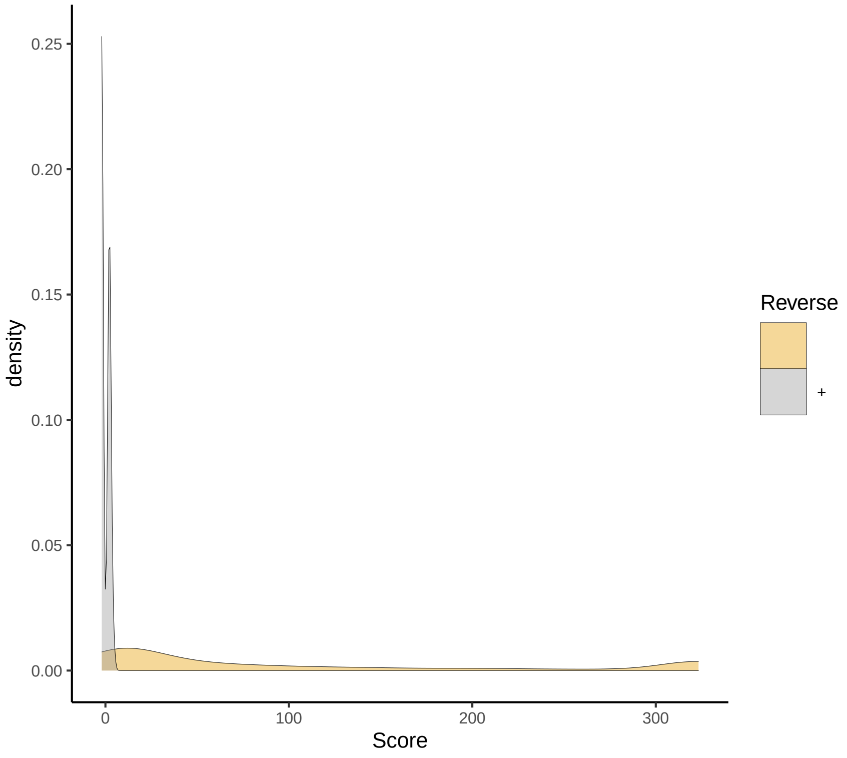

2.3 Initial Filtering

```
## Before filtering, proteinGroups.txt has 5366 rows (protein groups).
```

The subsequent initial filtering includes filtering out reverse hits as well as protein groups that were only identified by (modification) site.

```
## After initial filtering, 5138 rows (protein groups) remain.
```

2.4 Checking Normalization

2.4.1 Based on Boxplots

Plotting distributions of log2 raw intensities as well as log2 LFQ intensities for each sample:

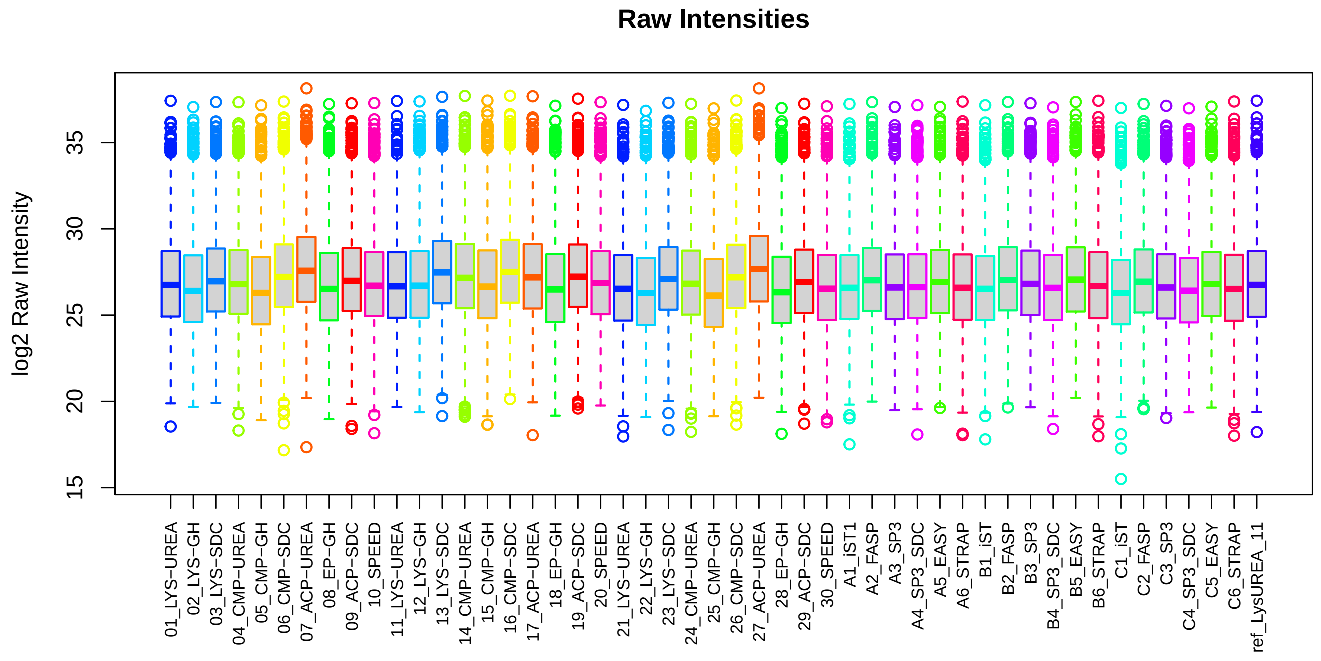

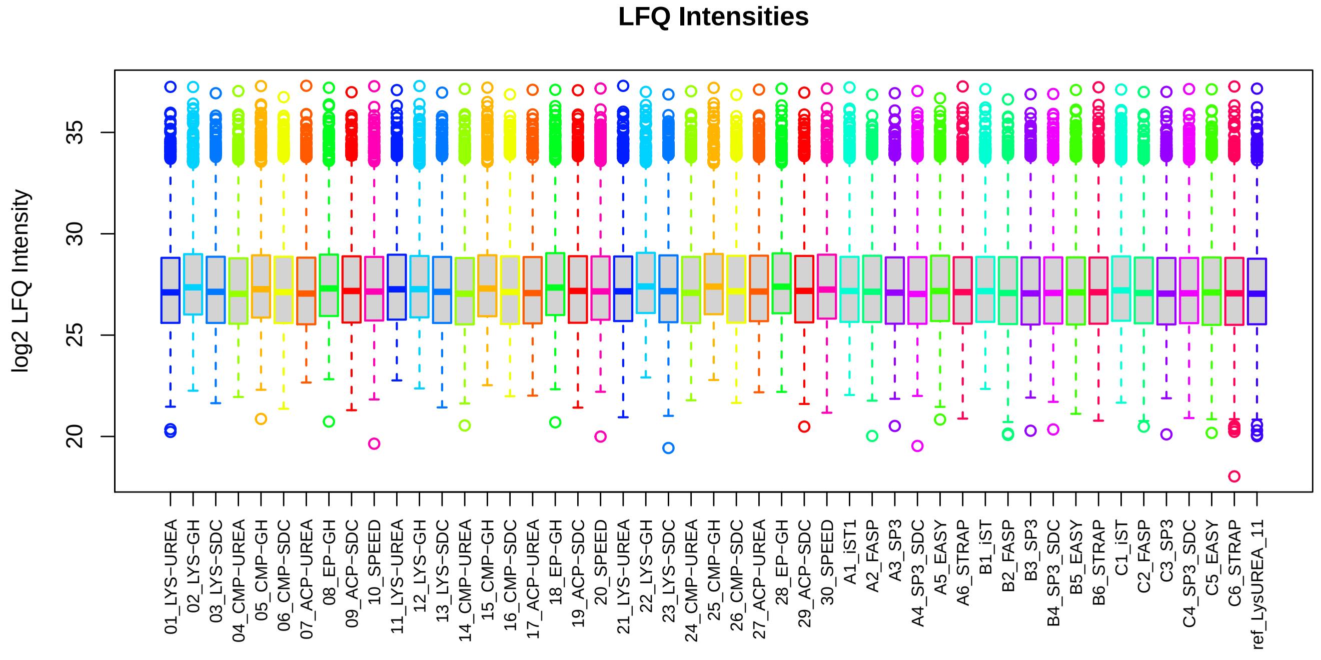

2.4.2 Based on Scatterplots

If there are more than 5 samples, the following plot will randomly select 5 samples and plot them against each other in their LFQ intensities:

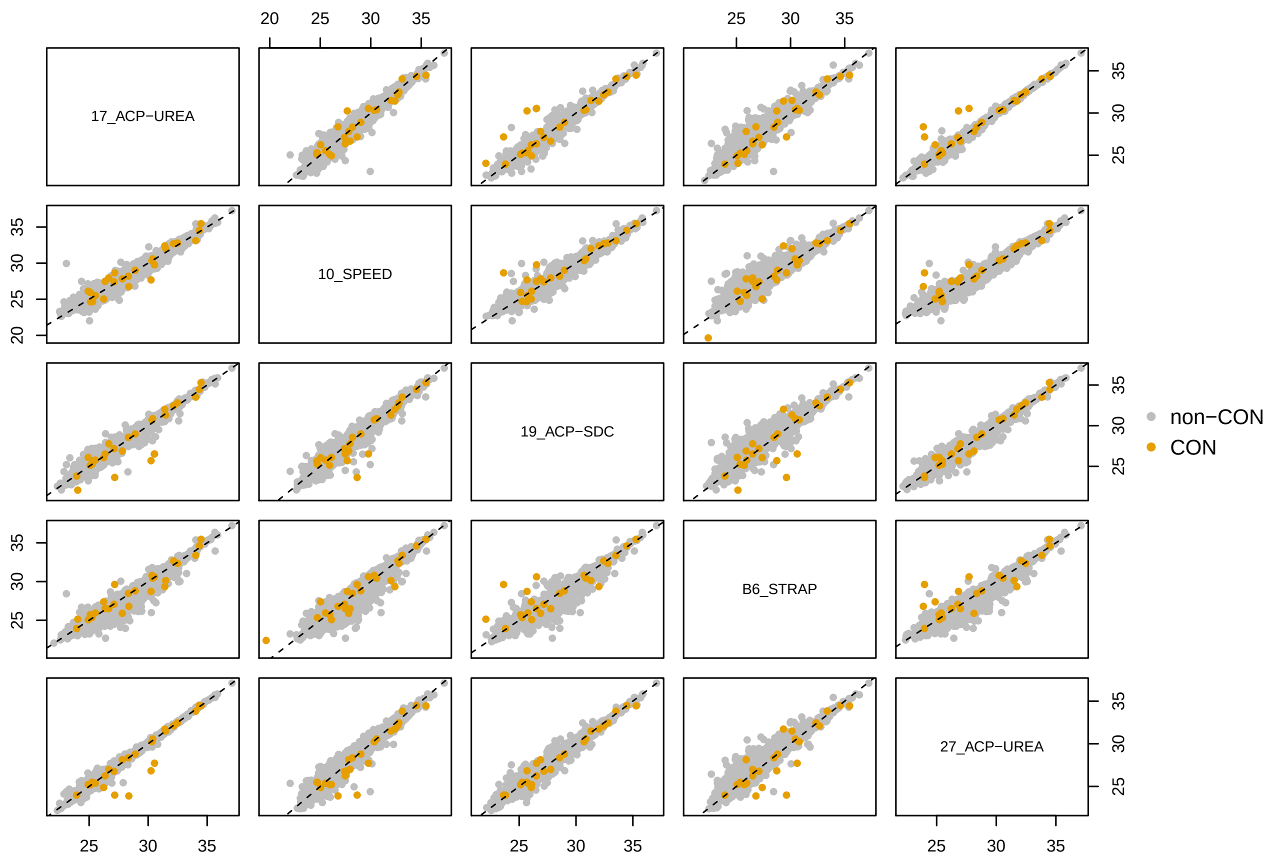

2.5 Checking Contamination and Top Proteins

Plotting relative amount of contaminants per sample (by dividing total contaminant iBAQ intensity by overall sample iBAQ intensity)

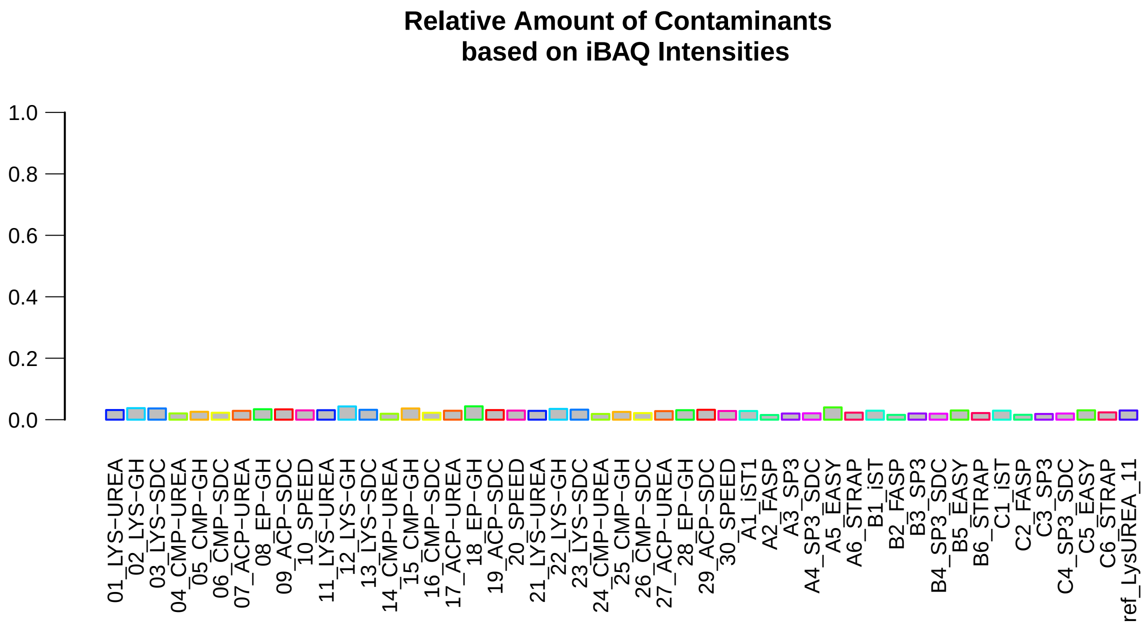

Listing the top protein groups (rows) of the whole experiment based on total iBAQ Intensities over all samples, including contaminants:

```
##         total_iBAQ Percentage      Name
## 1963 471415500000        3.6     H4C14
## 1859 425610300000        3.3      ACTB
## 1846 287572700000        2.2    H2BC13
## 2694 256817700000        2.0 HIST2H2AC
## 2036 254718400000        1.9      H3C2
## 768  245208900000        1.9     GAPDH
## 1984 182039700000        1.4      PPIA
## 97   151494800000        1.2      iRT9
## 2024 135838100000        1.0    EEF1A1
## 820  117058000000        0.9      NPM1
```

Taking a closer look at the following samples (per default: all samples):

```
##  [1] "09_ACP-SDC"      "19_ACP-SDC"      "29_ACP-SDC"      "07_ACP-UREA"
##  [5] "17_ACP-UREA"     "27_ACP-UREA"     "05_CMP-GH"       "15_CMP-GH"
##  [9] "25_CMP-GH"       "06_CMP-SDC"      "16_CMP-SDC"      "26_CMP-SDC"
## [13] "04_CMP-UREA"     "14_CMP-UREA"     "24_CMP-UREA"     "A5_EASY"
## [17] "B5_EASY"         "C5_EASY"         "08_EP-GH"        "18_EP-GH"
## [21] "28_EP-GH"        "A2_FASP"         "B2_FASP"         "C2_FASP"
## [25] "A1_iST1"         "B1_iST"          "C1_iST"          "02_LYS-GH"
## [29] "12_LYS-GH"       "22_LYS-GH"       "03_LYS-SDC"      "13_LYS-SDC"
## [33] "23_LYS-SDC"      "01_LYS-UREA"     "11_LYS-UREA"     "21_LYS-UREA"
## [37] "ref_LysUREA_11" "A3_SP3"          "B3_SP3"          "C3_SP3"
## [41] "A4_SP3_SDC"      "B4_SP3_SDC"      "C4_SP3_SDC"      "10_SPEED"
## [45] "20_SPEED"        "30_SPEED"        "A6_STRAP"        "B6_STRAP"
## [49] "C6_STRAP"
```

The following barplots show relative iBAQ intensities for each sample seperately. In each plot, the top x protein groups per sample including contaminants are highlighted. Per default, the top 10 protein groups + all other protein groups (aggregated to a single category ”other”, displayed in yellow) are shown, arranged in decreasing order from bottom to top - with the exception of ”other” proteins, which are always put at the very bottom.

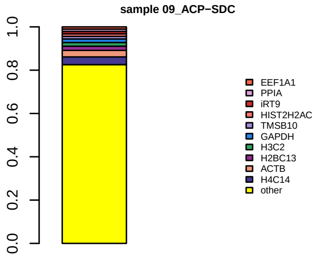

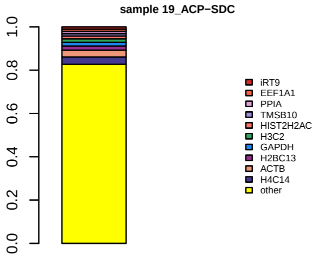

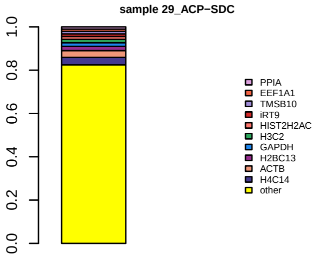

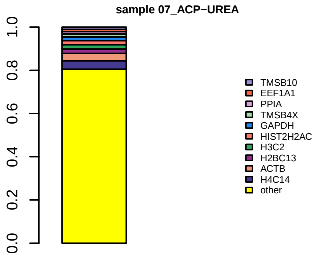

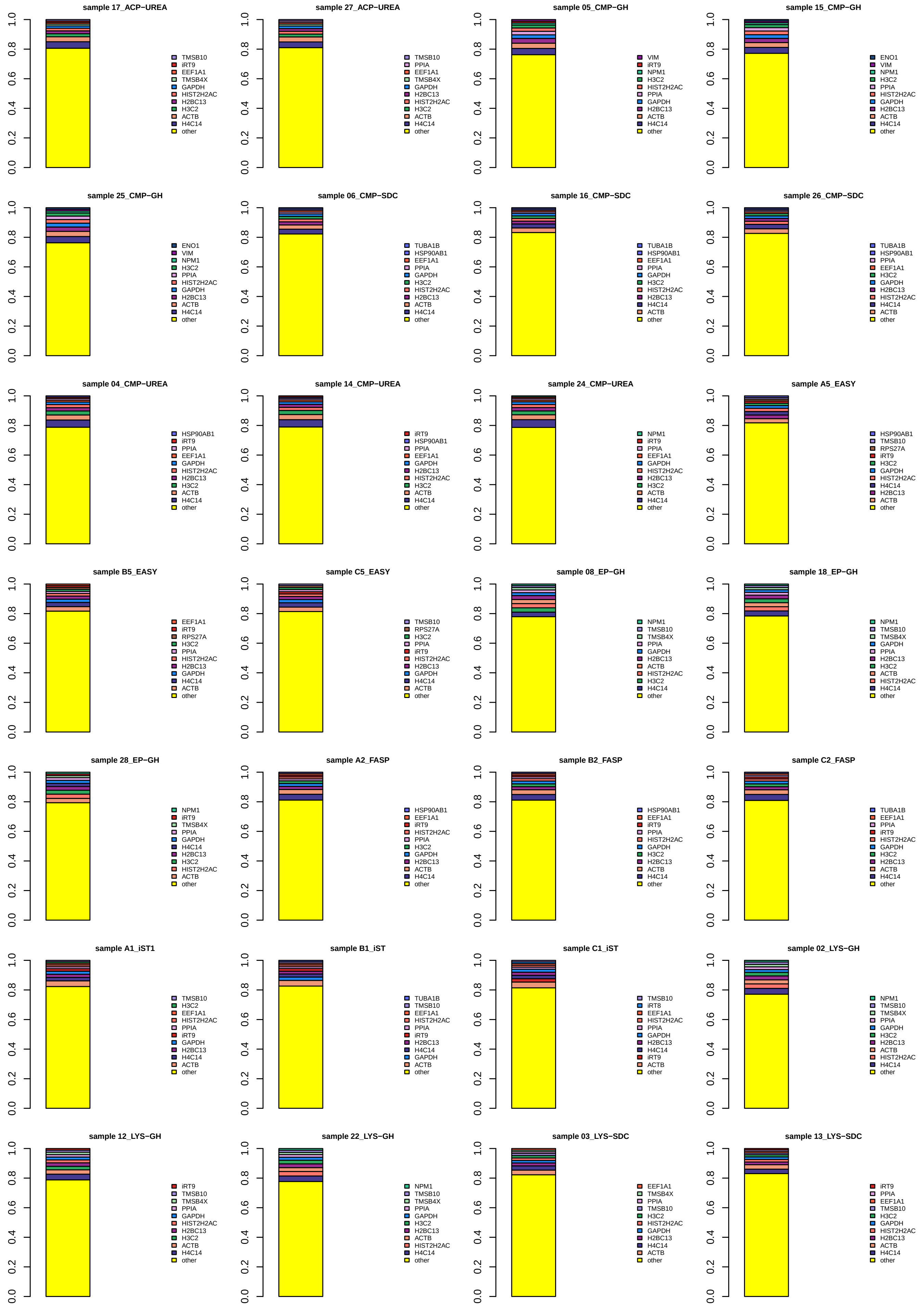
sample 17_ACP−UREA
TMSB10
iRT9
EEF1A1
TMSB4X
GAPDH
HIST2H2AC
H2BC13
H3C2
ACTB
H4C14
other
sample 27_ACP−UREA
TMSB10
PPIA
EEF1A1
TMSB4X
GAPDH
H2BC13
HIST2H2AC
H3C2
ACTB
H4C14
other
sample 05_CMP−GH
VIM
iRT9
NPM1
H3C2
HIST2H2AC
PPIA
GAPDH
H2BC13
ACTB
H4C14
other
sample 15_CMP−GH
ENO1
VIM
NPM1
H3C2
PPIA
HIST2H2AC
GAPDH
H2BC13
ACTB
H4C14
other
sample 25_CMP−GH
ENO1
VIM
NPM1
H3C2
PPIA
HIST2H2AC
GAPDH
H2BC13
ACTB
H4C14
other
sample 06_CMP−SDC
TUBA1B
HSP90AB1
EEF1A1
PPIA
GAPDH
H3C2
HIST2H2AC
H2BC13
ACTB
H4C14
other
sample 16_CMP−SDC
TUBA1B
HSP90AB1
EEF1A1
PPIA
GAPDH
H3C2
HIST2H2AC
H2BC13
H4C14
ACTB
other
sample 26_CMP−SDC
TUBA1B
HSP90AB1
PPIA
EEF1A1
H3C2
GAPDH
H2BC13
HIST2H2AC
H4C14
ACTB
other
sample 04_CMP−UREA
HSP90AB1
iRT9
PPIA
EEF1A1
GAPDH
HIST2H2AC
H2BC13
H3C2
ACTB
H4C14
other
sample 14_CMP−UREA
iRT9
HSP90AB1
PPIA
EEF1A1
GAPDH
H2BC13
HIST2H2AC
H3C2
ACTB
H4C14
other
sample 24_CMP−UREA
NPM1
iRT9
PPIA
EEF1A1
GAPDH
HIST2H2AC
H2BC13
H3C2
ACTB
H4C14
other
sample A5_EASY
HSP90AB1
TMSB10
RPS27A
iRT9
H3C2
GAPDH
HIST2H2AC
H4C14
H2BC13
ACTB
other
sample B5_EASY
EEF1A1
iRT9
RPS27A
H3C2
PPIA
HIST2H2AC
H2BC13
GAPDH
H4C14
ACTB
other
sample C5_EASY
TMSB10
RPS27A
H3C2
PPIA
iRT9
HIST2H2AC
H2BC13
GAPDH
H4C14
ACTB
other
sample 08_EP−GH
NPM1
TMSB10
TMSB4X
PPIA
GAPDH
H2BC13
ACTB
HIST2H2AC
H3C2
H4C14
other
sample 18_EP−GH
NPM1
TMSB10
TMSB4X
GAPDH
PPIA
H2BC13
H3C2
ACTB
HIST2H2AC
H4C14
other
sample 28_EP−GH
NPM1
iRT9
TMSB4X
PPIA
GAPDH
H4C14
H2BC13
H3C2
HIST2H2AC
ACTB
other
sample A2_FASP
HSP90AB1
EEF1A1
iRT9
HIST2H2AC
PPIA
H3C2
GAPDH
H2BC13
ACTB
H4C14
other
sample B2_FASP
HSP90AB1
EEF1A1
iRT9
PPIA
HIST2H2AC
GAPDH
H3C2
H2BC13
ACTB
H4C14
other
sample C2_FASP
TUBA1B
EEF1A1
PPIA
iRT9
HIST2H2AC
GAPDH
H3C2
H2BC13
ACTB
H4C14
other
sample A1_iST1
TMSB10
H3C2
EEF1A1
HIST2H2AC
PPIA
iRT9
GAPDH
H2BC13
H4C14
ACTB
other
sample B1_iST
TUBA1B
TMSB10
EEF1A1
HIST2H2AC
PPIA
iRT9
H2BC13
H4C14
GAPDH
ACTB
other
sample C1_iST
TMSB10
iRT8
EEF1A1
HIST2H2AC
PPIA
GAPDH
H2BC13
H4C14
iRT9
ACTB
other
sample 02_LYS−GH
NPM1
TMSB10
TMSB4X
PPIA
GAPDH
H3C2
H2BC13
ACTB
HIST2H2AC
H4C14
other
sample 12_LYS−GH
iRT9
TMSB10
TMSB4X
PPIA
GAPDH
HIST2H2AC
H2BC13
H3C2
ACTB
H4C14
other
sample 22_LYS−GH
NPM1
TMSB10
TMSB4X
PPIA
GAPDH
H3C2
H2BC13
ACTB
HIST2H2AC
H4C14
other
sample 03_LYS−SDC
EEF1A1
TMSB4X
PPIA
TMSB10
H3C2
HIST2H2AC
GAPDH
H2BC13
H4C14
ACTB
other
sample 13_LYS−SDC
iRT9
PPIA
EEF1A1
TMSB10
H3C2
GAPDH
HIST2H2AC
H2BC13
ACTB
H4C14
other

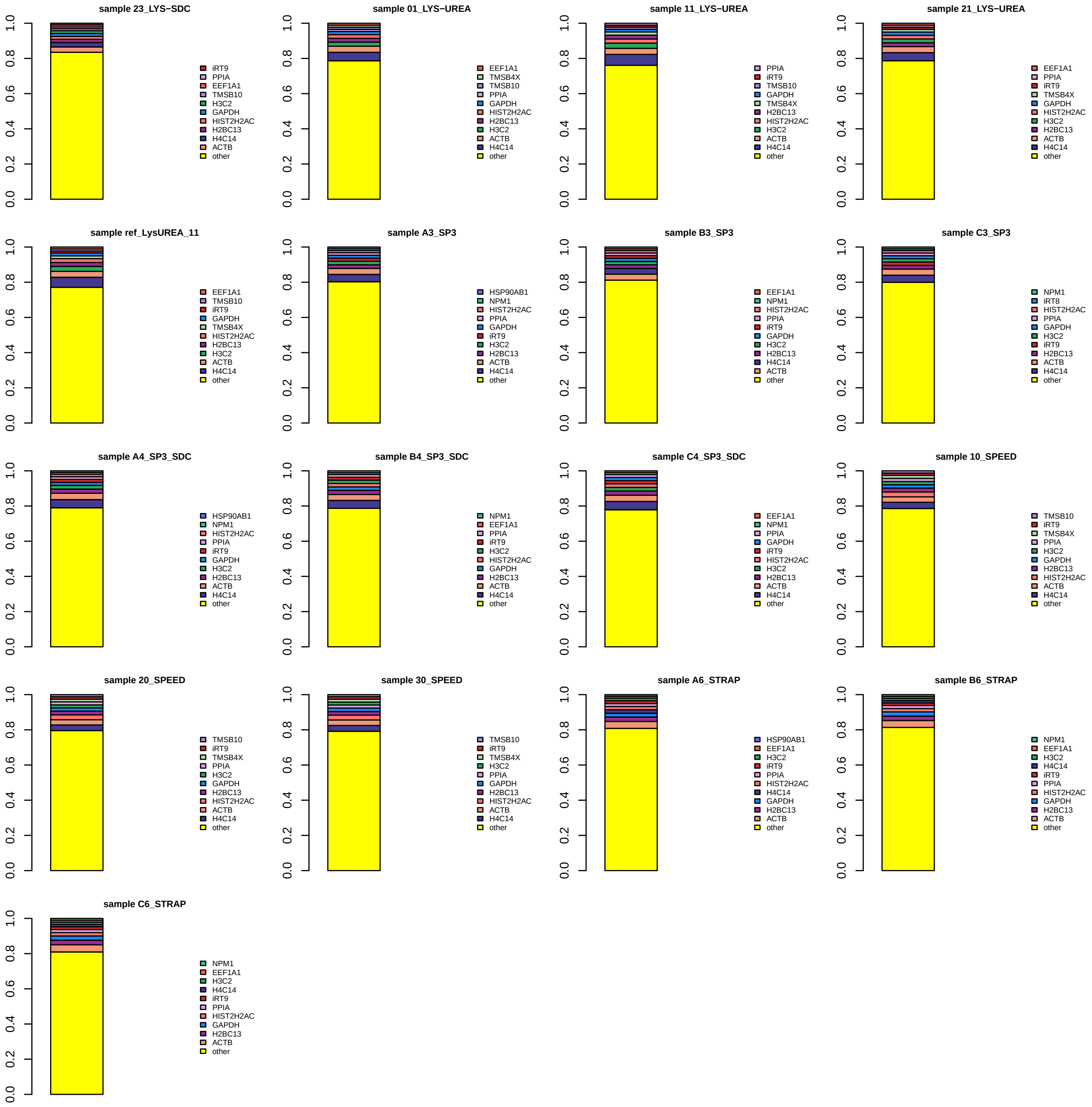

The following barplots show the relative amount of the top x contaminants based on all Contaminants (therefore always scaling up to 1!), for each sample seperately.

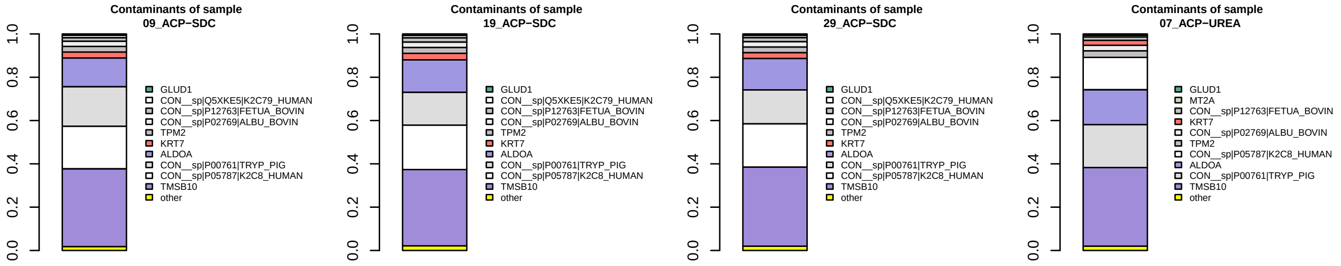

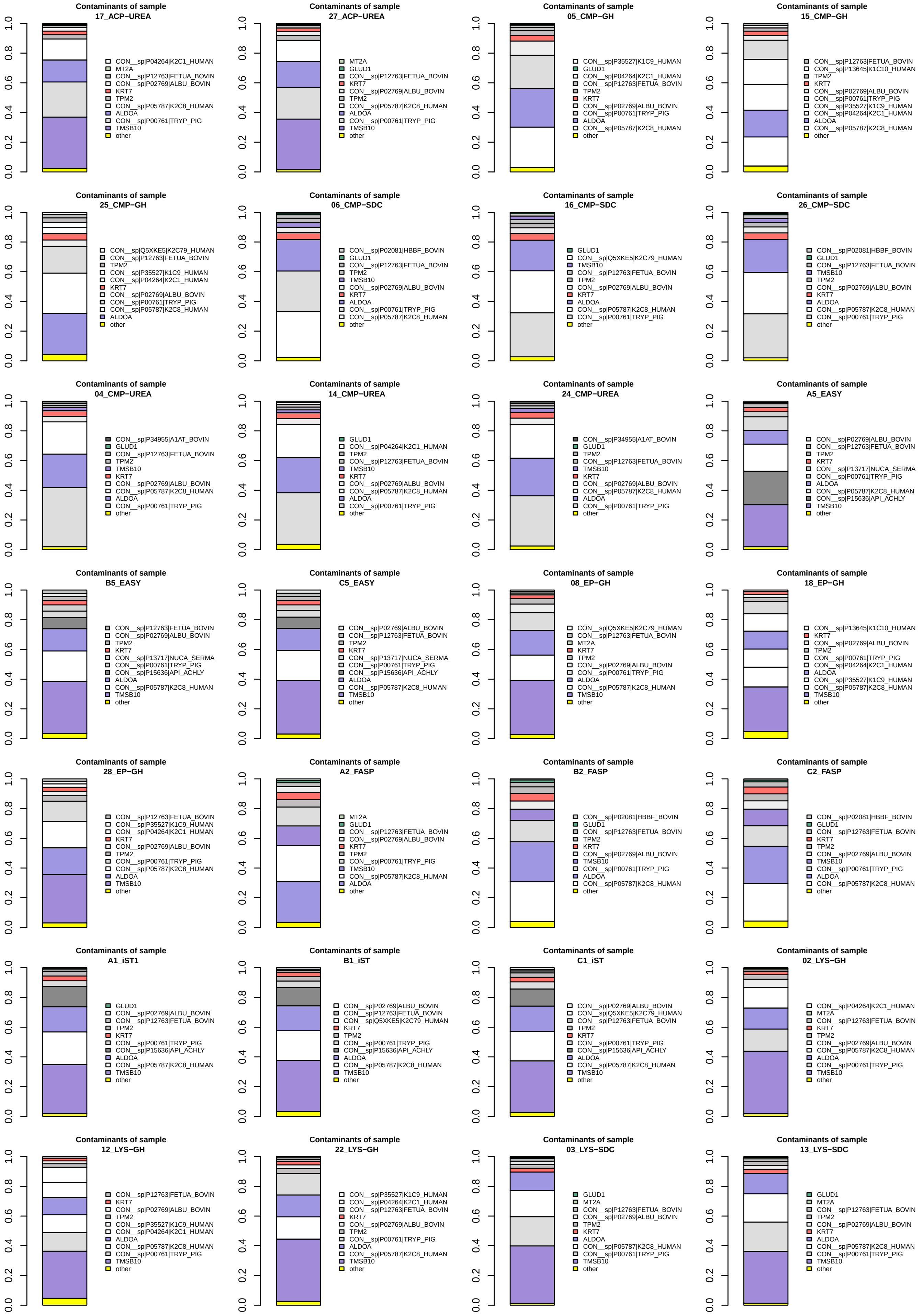

Contaminants of sample 17_ACP−UREA
CON__sp|P04264|K2C1_HUMAN
MT2A
CON__sp|P12763|FETUA_BOVIN
CON__sp|P02769|ALBU_BOVIN
KRT7
TPM2
CON__sp|P05787|K2C8_HUMAN
ALDOA
CON__sp|P00761|TRYP_PIG
TMSB10
other
Contaminants of sample 27_ACP−UREA
MT2A
GLUD1
CON__sp|P12763|FETUA_BOVIN
KRT7
CON__sp|P02769|ALBU_BOVIN
TPM2
CON__sp|P05787|K2C8_HUMAN
ALDOA
CON__sp|P00761|TRYP_PIG
TMSB10
other
Contaminants of sample 05_CMP−GH
CON__sp|P35527|K1C9_HUMAN
GLUD1
CON__sp|P04264|K2C1_HUMAN
CON__sp|P12763|FETUA_BOVIN
TPM2
KRT7
CON__sp|P02769|ALBU_BOVIN
CON__sp|P00761|TRYP_PIG
ALDOA
CON__sp|P05787|K2C8_HUMAN
other
Contaminants of sample 15_CMP−GH
CON__sp|P12763|FETUA_BOVIN
CON__sp|P13645|K1C10_HUMAN
TPM2
KRT7
CON__sp|P02769|ALBU_BOVIN
CON__sp|P00761|TRYP_PIG
CON__sp|P35527|K1C9_HUMAN
CON__sp|P04264|K2C1_HUMAN
ALDOA
CON__sp|P05787|K2C8_HUMAN
other
Contaminants of sample 25_CMP−GH
CON__sp|Q5XKE5|K2C79_HUMAN
CON__sp|P12763|FETUA_BOVIN
TPM2
CON__sp|P35527|K1C9_HUMAN
CON__sp|P04264|K2C1_HUMAN
KRT7
CON__sp|P02769|ALBU_BOVIN
CON__sp|P00761|TRYP_PIG
CON__sp|P05787|K2C8_HUMAN
ALDOA
other
Contaminants of sample 06_CMP−SDC
CON__sp|P02081|HBBF_BOVIN
GLUD1
CON__sp|P12763|FETUA_BOVIN
TPM2
TMSB10
CON__sp|P02769|ALBU_BOVIN
KRT7
ALDOA
CON__sp|P00761|TRYP_PIG
CON__sp|P05787|K2C8_HUMAN
other
Contaminants of sample 16_CMP−SDC
GLUD1
CON__sp|Q5XKE5|K2C79_HUMAN
TMSB10
CON__sp|P12763|FETUA_BOVIN
TPM2
CON__sp|P02769|ALBU_BOVIN
KRT7
ALDOA
CON__sp|P05787|K2C8_HUMAN
CON__sp|P00761|TRYP_PIG
other
Contaminants of sample 26_CMP−GH
CON__sp|P02081|HBBF_BOVIN
GLUD1
CON__sp|P12763|FETUA_BOVIN
TMSB10
TPM2
CON__sp|P02769|ALBU_BOVIN
KRT7
ALDOA
CON__sp|P05787|K2C8_HUMAN
CON__sp|P00761|TRYP_PIG
other
Contaminants of sample 04_CMP−UREA
CON__sp|P34955|A1AT_BOVIN
GLUD1
CON__sp|P12763|FETUA_BOVIN
TPM2
TMSB10
KRT7
CON__sp|P02769|ALBU_BOVIN
CON__sp|P05787|K2C8_HUMAN
ALDOA
CON__sp|P00761|TRYP_PIG
other
Contaminants of sample 14_CMP−UREA
GLUD1
CON__sp|P04264|K2C1_HUMAN
TPM2
CON__sp|P12763|FETUA_BOVIN
TMSB10
KRT7
CON__sp|P02769|ALBU_BOVIN
CON__sp|P05787|K2C8_HUMAN
ALDOA
CON__sp|P00761|TRYP_PIG
other
Contaminants of sample 24_CMP−UREA
CON__sp|P34955|A1AT_BOVIN
GLUD1
TPM2
CON__sp|P12763|FETUA_BOVIN
TMSB10
KRT7
CON__sp|P02769|ALBU_BOVIN
CON__sp|P05787|K2C8_HUMAN
ALDOA
CON__sp|P00761|TRYP_PIG
other
Contaminants of sample A5_EASY
CON__sp|P02769|ALBU_BOVIN
CON__sp|P12763|FETUA_BOVIN
TPM2
KRT7
CON__sp|P13717|NUCA_SERMA
CON__sp|P00761|TRYP_PIG
ALDOA
CON__sp|P05787|K2C8_HUMAN
CON__sp|P15636|API_ACHLY
TMSB10
other
Contaminants of sample B5_EASY
CON__sp|P12763|FETUA_BOVIN
CON__sp|P02769|ALBU_BOVIN
TPM2
KRT7
CON__sp|P13717|NUCA_SERMA
CON__sp|P00761|TRYP_PIG
CON__sp|P15636|API_ACHLY
ALDOA
CON__sp|P05787|K2C8_HUMAN
TMSB10
other
Contaminants of sample C5_EASY
CON__sp|P02769|ALBU_BOVIN
CON__sp|P12763|FETUA_BOVIN
TPM2
KRT7
CON__sp|P13717|NUCA_SERMA
CON__sp|P00761|TRYP_PIG
CON__sp|P15636|API_ACHLY
ALDOA
CON__sp|P05787|K2C8_HUMAN
TMSB10
other
Contaminants of sample 08_EP−GH
CON__sp|Q5XKE5|K2C79_HUMAN
CON__sp|P12763|FETUA_BOVIN
MT2A
KRT7
TPM2
CON__sp|P02769|ALBU_BOVIN
CON__sp|P00761|TRYP_PIG
ALDOA
CON__sp|P05787|K2C8_HUMAN
TMSB10
other
Contaminants of sample 18_EP−GH
CON__sp|P13645|K1C10_HUMAN
KRT7
CON__sp|P02769|ALBU_BOVIN
TPM2
CON__sp|P00761|TRYP_PIG
CON__sp|P04264|K2C1_HUMAN
ALDOA
CON__sp|P35527|K1C9_HUMAN
CON__sp|P05787|K2C8_HUMAN
TMSB10
other
Contaminants of sample 28_EP−GH
CON__sp|P12763|FETUA_BOVIN
CON__sp|P35527|K1C9_HUMAN
CON__sp|P04264|K2C1_HUMAN
KRT7
CON__sp|P02769|ALBU_BOVIN
TPM2
CON__sp|P00761|TRYP_PIG
CON__sp|P05787|K2C8_HUMAN
ALDOA
TMSB10
other
Contaminants of sample A2_FASP
MT2A
GLUD1
CON__sp|P12763|FETUA_BOVIN
CON__sp|P02769|ALBU_BOVIN
KRT7
TPM2
CON__sp|P00761|TRYP_PIG
TMSB10
CON__sp|P05787|K2C8_HUMAN
ALDOA
other
Contaminants of sample B2_FASP
CON__sp|P02081|HBBF_BOVIN
GLUD1
CON__sp|P12763|FETUA_BOVIN
TPM2
KRT7
CON__sp|P02769|ALBU_BOVIN
TMSB10
CON__sp|P00761|TRYP_PIG
ALDOA
CON__sp|P05787|K2C8_HUMAN
other
Contaminants of sample C2_FASP
CON__sp|P02081|HBBF_BOVIN
GLUD1
CON__sp|P12763|FETUA_BOVIN
KRT7
TPM2
CON__sp|P02769|ALBU_BOVIN
TMSB10
CON__sp|P00761|TRYP_PIG
ALDOA
CON__sp|P05787|K2C8_HUMAN
other
Contaminants of sample A1_iST1
GLUD1
CON__sp|P02769|ALBU_BOVIN
CON__sp|P12763|FETUA_BOVIN
TPM2
KRT7
CON__sp|P00761|TRYP_PIG
CON__sp|P15636|API_ACHLY
ALDOA
CON__sp|P05787|K2C8_HUMAN
TMSB10
other
Contaminants of sample B1_iST
CON__sp|P02769|ALBU_BOVIN
CON__sp|P12763|FETUA_BOVIN
CON__sp|Q5XKE5|K2C79_HUMAN
KRT7
TPM2
CON__sp|P00761|TRYP_PIG
CON__sp|P15636|API_ACHLY
ALDOA
CON__sp|P05787|K2C8_HUMAN
TMSB10
other
Contaminants of sample C1_iST
CON__sp|P02769|ALBU_BOVIN
CON__sp|Q5XKE5|K2C79_HUMAN
CON__sp|P12763|FETUA_BOVIN
TPM2
KRT7
CON__sp|P00761|TRYP_PIG
CON__sp|P15636|API_ACHLY
ALDOA
CON__sp|P05787|K2C8_HUMAN
TMSB10
other
Contaminants of sample 02_LYS−GH
CON__sp|P04264|K2C1_HUMAN
MT2A
CON__sp|P12763|FETUA_BOVIN
KRT7
TPM2
CON__sp|P02769|ALBU_BOVIN
CON__sp|P05787|K2C8_HUMAN
ALDOA
CON__sp|P00761|TRYP_PIG
TMSB10
other
Contaminants of sample 12_LYS−GH
CON__sp|P12763|FETUA_BOVIN
KRT7
CON__sp|P02769|ALBU_BOVIN
TPM2
CON__sp|P35527|K1C9_HUMAN
CON__sp|P04264|K2C1_HUMAN
ALDOA
CON__sp|P05787|K2C8_HUMAN
CON__sp|P00761|TRYP_PIG
TMSB10
other
Contaminants of sample 22_LYS−GH
CON__sp|P35527|K1C9_HUMAN
CON__sp|P04264|K2C1_HUMAN
CON__sp|P12763|FETUA_BOVIN
KRT7
CON__sp|P02769|ALBU_BOVIN
TPM2
CON__sp|P00761|TRYP_PIG
ALDOA
CON__sp|P05787|K2C8_HUMAN
TMSB10
other
Contaminants of sample 03_LYS−SDC
GLUD1
MT2A
CON__sp|P12763|FETUA_BOVIN
CON__sp|P02769|ALBU_BOVIN
TPM2
KRT7
ALDOA
CON__sp|P05787|K2C8_HUMAN
CON__sp|P00761|TRYP_PIG
TMSB10
other
Contaminants of sample 13_LYS−SDC
GLUD1
MT2A
CON__sp|P12763|FETUA_BOVIN
TPM2
CON__sp|P02769|ALBU_BOVIN
KRT7
ALDOA
CON__sp|P05787|K2C8_HUMAN
CON__sp|P00761|TRYP_PIG
TMSB10
other

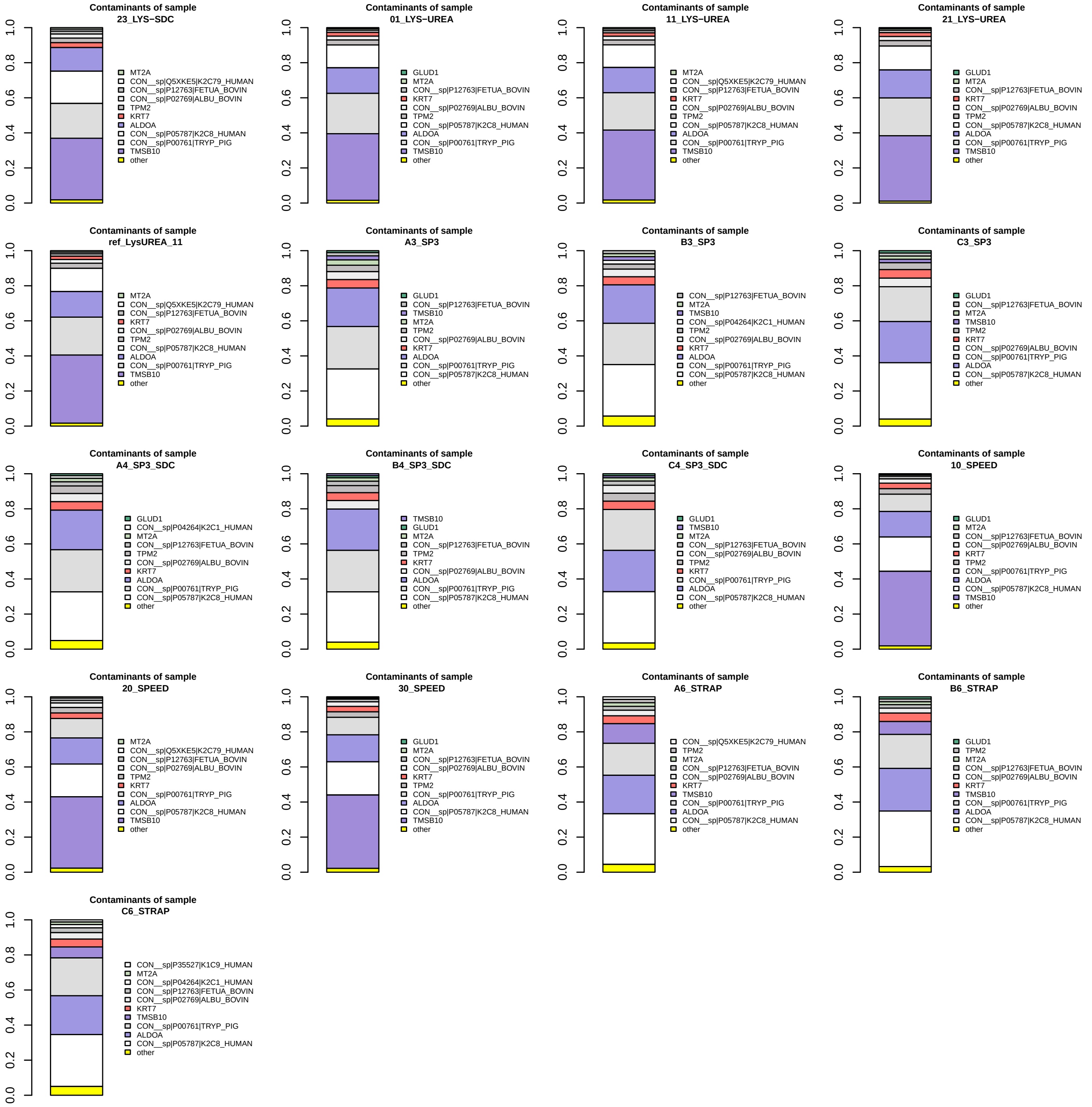

3 Advanced Filtering

3.1 Based on Contaminants

This filtering step filters out rows (protein groups) considered as contaminants, as long as the respective parameter is set on TRUE (default setting). The current parameter chosen is:

```
print(remove_contaminants)

## [1] TRUE
```

```
## Before this filtering step, there are  5138 rows (protein groups).
## After this filtering step, 5095 rows (protein groups) remain.
```

3.2 Based on Razor + Unique Peptides

```r
print(razor_plus_unique_peptides_filter)

## [1] TRUE
```

```
## Before this filtering step, there are  5095 rows (protein groups).
## Removing rows (protein groups) with less than 2 razor + unique peptides.
## After this filtering step, 4883 rows (protein groups) remain.
```

3.3 Based on Valid Values

This final filtering step filters out rows (protein groups) based on minimum number of valid values in the LFQ intensity columns (in case a renormalization stragegy is employed, this filtering step is instead based on the minimum number of valid values in the raw intensity columns). The mode and the minimum number of valid values can be changed via their corresponding parameters. The parameters currently chosen are:

```r
print(mode_valid_values_filter)

## [1] "in_at_least_one_group"

print(number_valid_values_filter)

## [1] 3
```

```
## Before this filtering step, there are  4883 rows (protein groups).
## After this filtering step, 4263 rows (protein groups) remain.
```

The rest of this report will focus solely on the proteins (rows) that are left after this final filtering step, i.e. every protein that has been discarded by now will not be included in the subsequent analysis.

3.4 Renormalization after Advanced Filtering

All the available renormalization methods use the raw intensities only. Choosing one will replace the MaxQuant LFQ intensities with normalized raw intensities (i.e. new LFQ intensities are created and used for the remainder of the analysis).

```r
print(renormalization_median)

## [1] FALSE

print(renormalization_quantile)
```

```
## [1] FALSE

print(renormalization_loess)

## [1] FALSE

print(renormalization_to_proteins)

## NULL

print(renormalization_to_sample)

## NULL
```

4 Visualization before Imputation

4.1 Remaining Missing Values

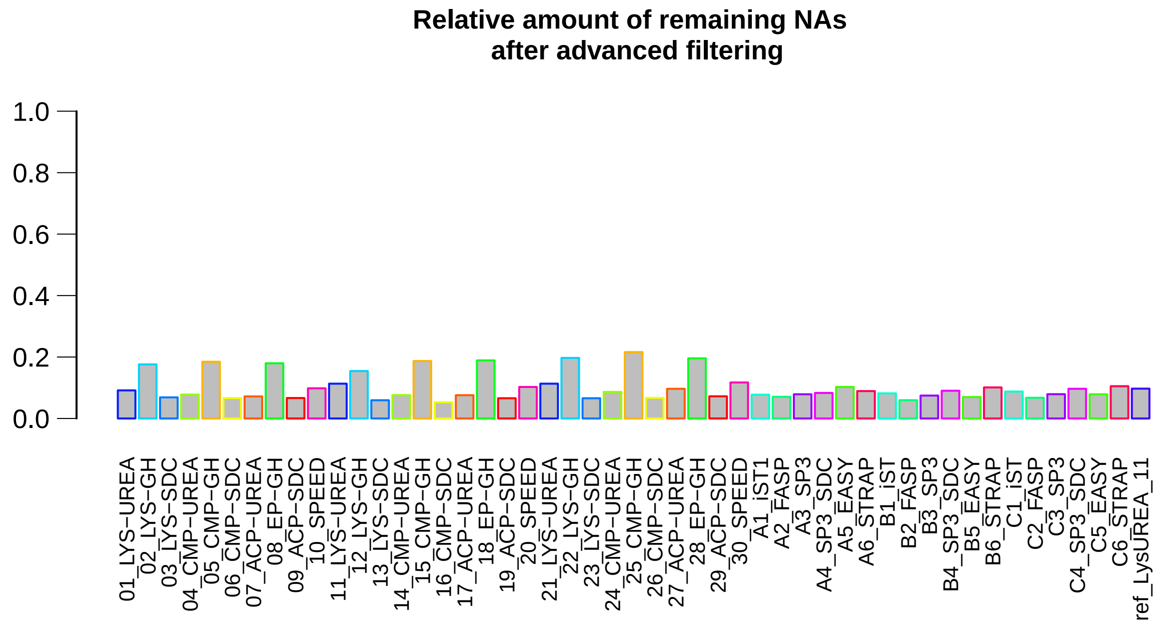

4.2 Heatmap before Imputation

The following plot is based on log2 LFQ-intensities of the remaining protein groups (rows), with missing data being set to 0. Blue represents low intensities (in this case 0), and increasingly reddish color signifies higher intensities.

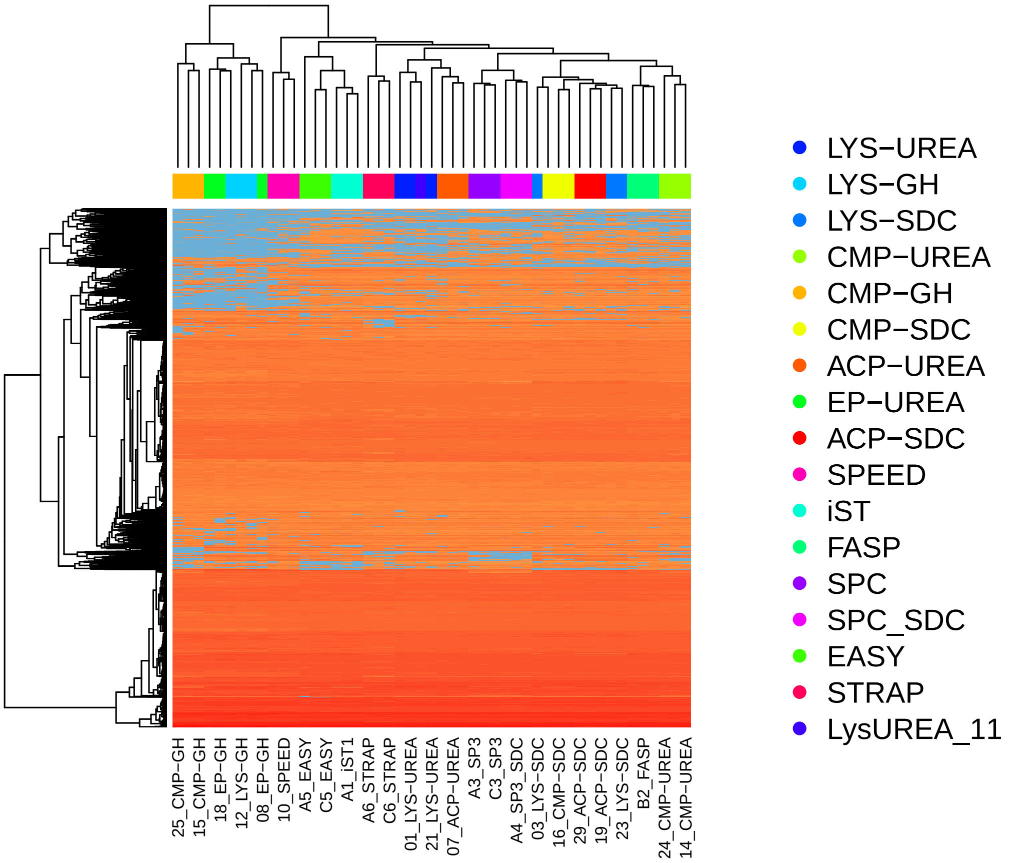

4.3 PCA before Imputation

This plot is based on log2 LFQ-intensities of the remaining proteins (rows), with missing data being set to 0:

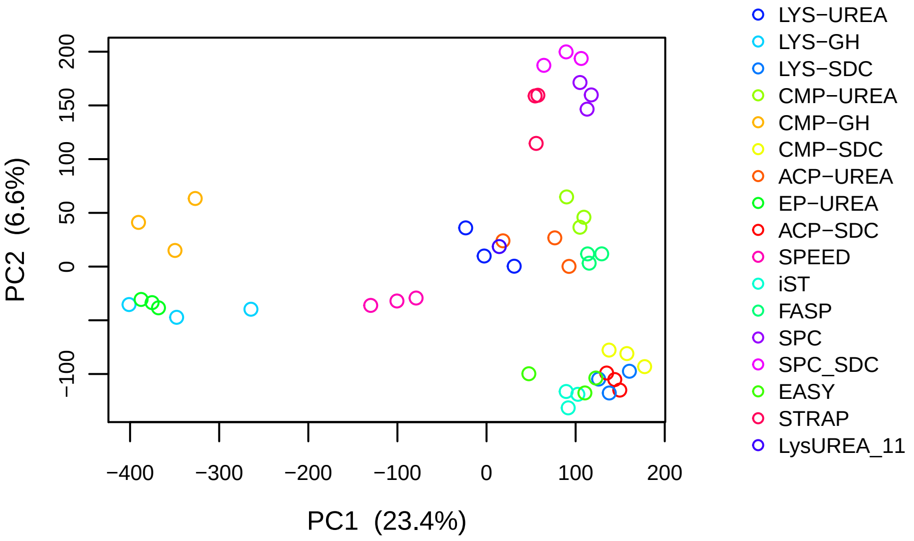

5 Imputation of Missing Values

In the next step, missing values will be imputed - either by a constant that equals the minimal log2 LFQ intensity over all samples, rounded down; or by a downshifted normal distribution. The mode of imputation can be changed via the respective parameter, the current parameter being:

```
print(mode_imputation)

## [1] "constant"
```

```
## Before doing imputation, there are 21213 missing intensity values.
## The constant value that is used for imputation was calculated to be 21
```

```
## After doing imputation, 0 missing intensity values remain.
```

Plotting the distribution of log2 intensities before and after imputation for each sample:

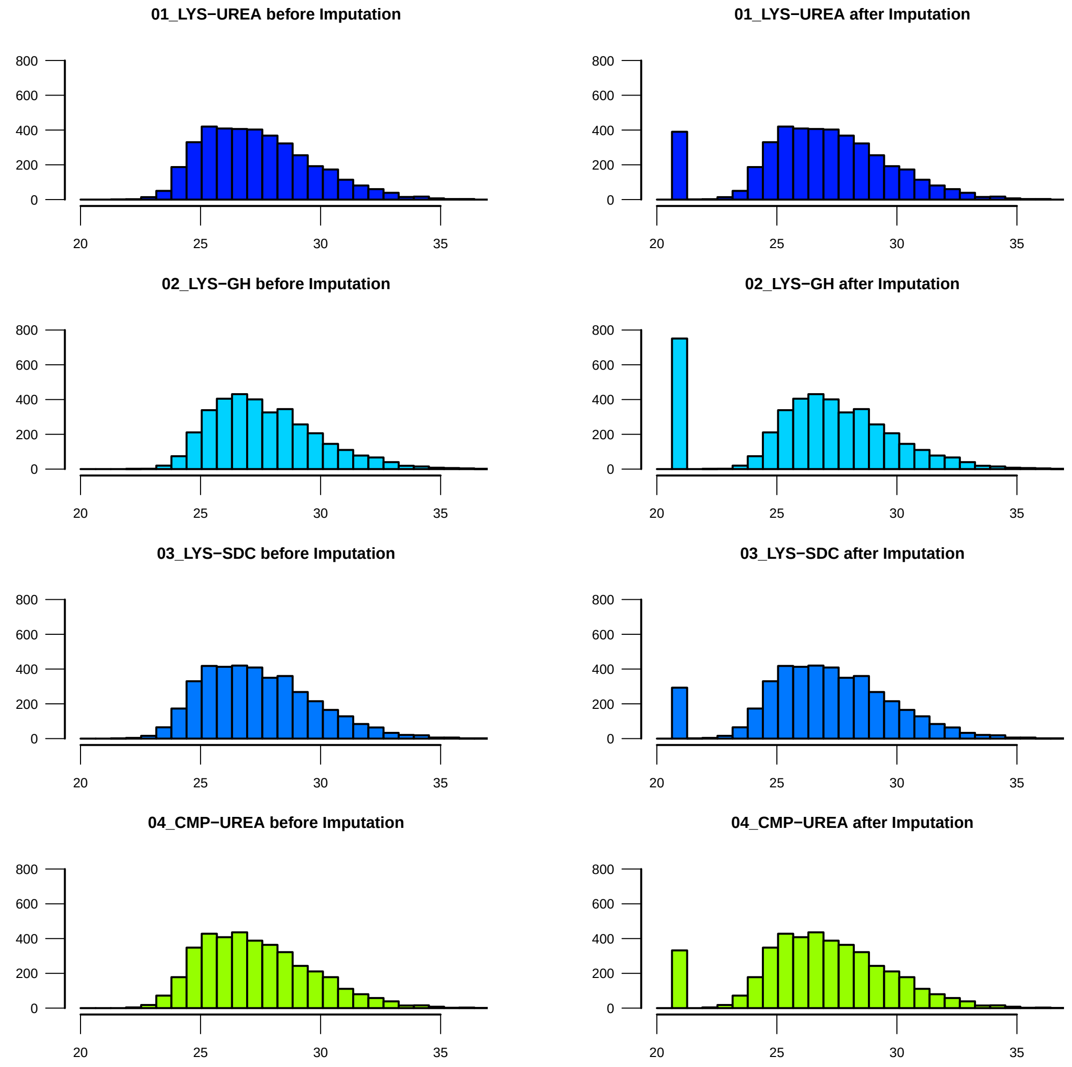

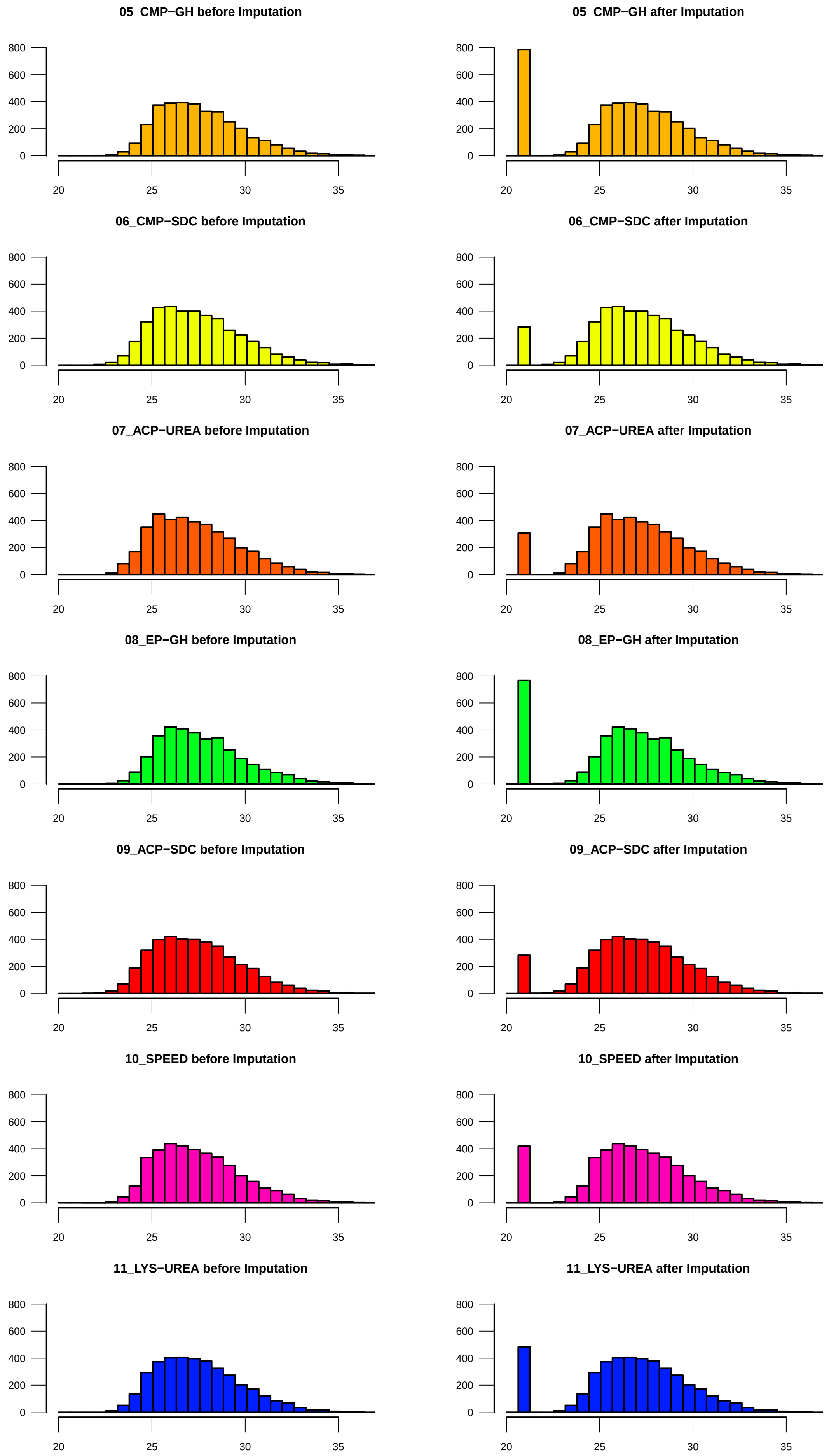
05_CMP−GH before Imputation
05_CMP−GH after Imputation
06_CMP−SDC before Imputation
06_CMP−SDC after Imputation
07_ACP−UREA before Imputation
07_ACP−UREA after Imputation
08_EP−GH before Imputation
08_EP−GH after Imputation
09_ACP−SDC before Imputation
09_ACP−SDC after Imputation
10_SPEED before Imputation
10_SPEED after Imputation
11_LYS−UREA before Imputation
11_LYS−UREA after Imputation

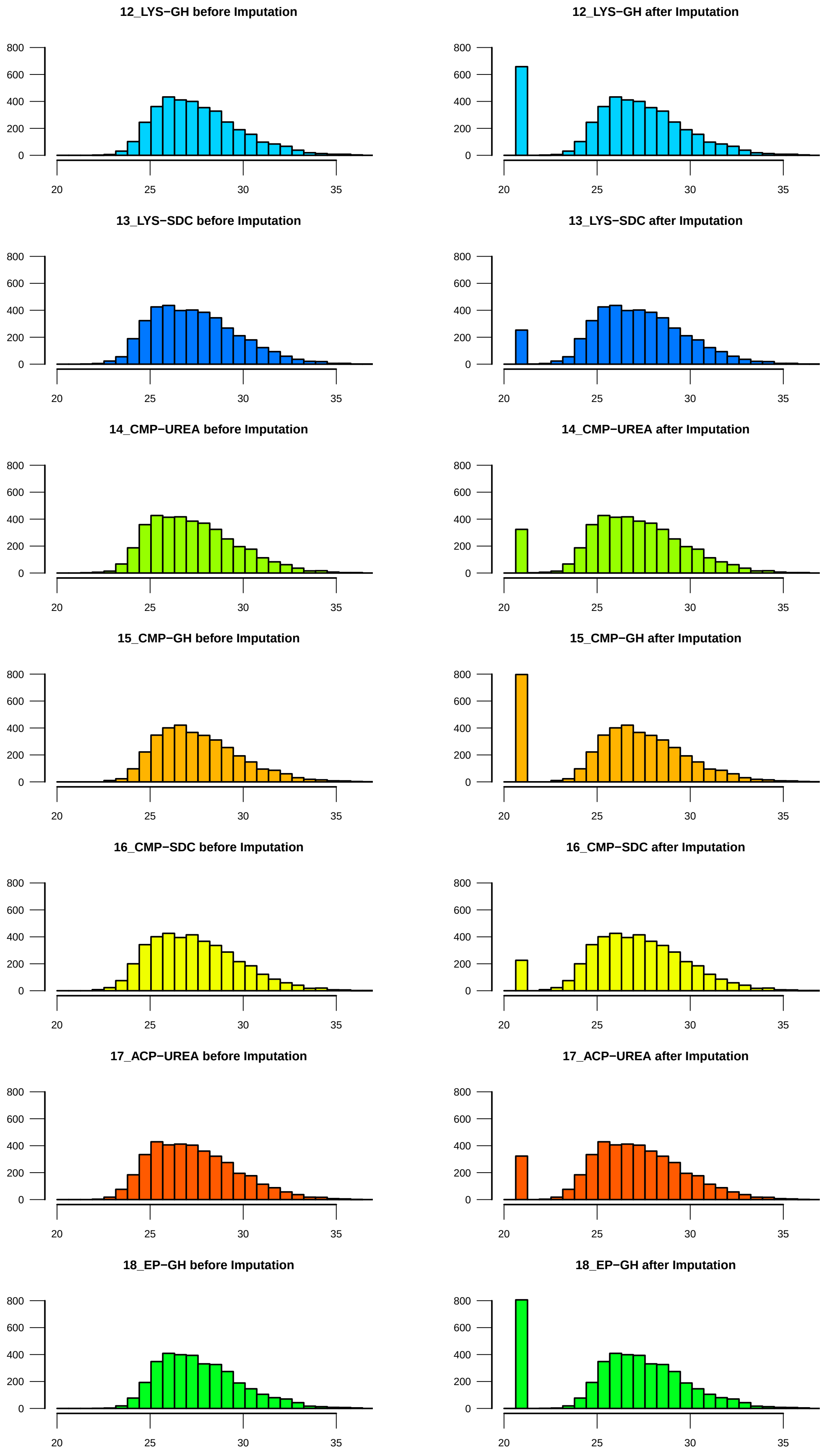
12_LYS−GH before Imputation
12_LYS−GH after Imputation
13_LYS−SDC before Imputation
13_LYS−SDC after Imputation
14_CMP−UREA before Imputation
14_CMP−UREA after Imputation
15_CMP−GH before Imputation
15_CMP−GH after Imputation
16_CMP−SDC before Imputation
16_CMP−SDC after Imputation
17_ACP−UREA before Imputation
17_ACP−UREA after Imputation
18_EP−GH before Imputation
18_EP−GH after Imputation
0
200
400
600
800
20
25
30
35

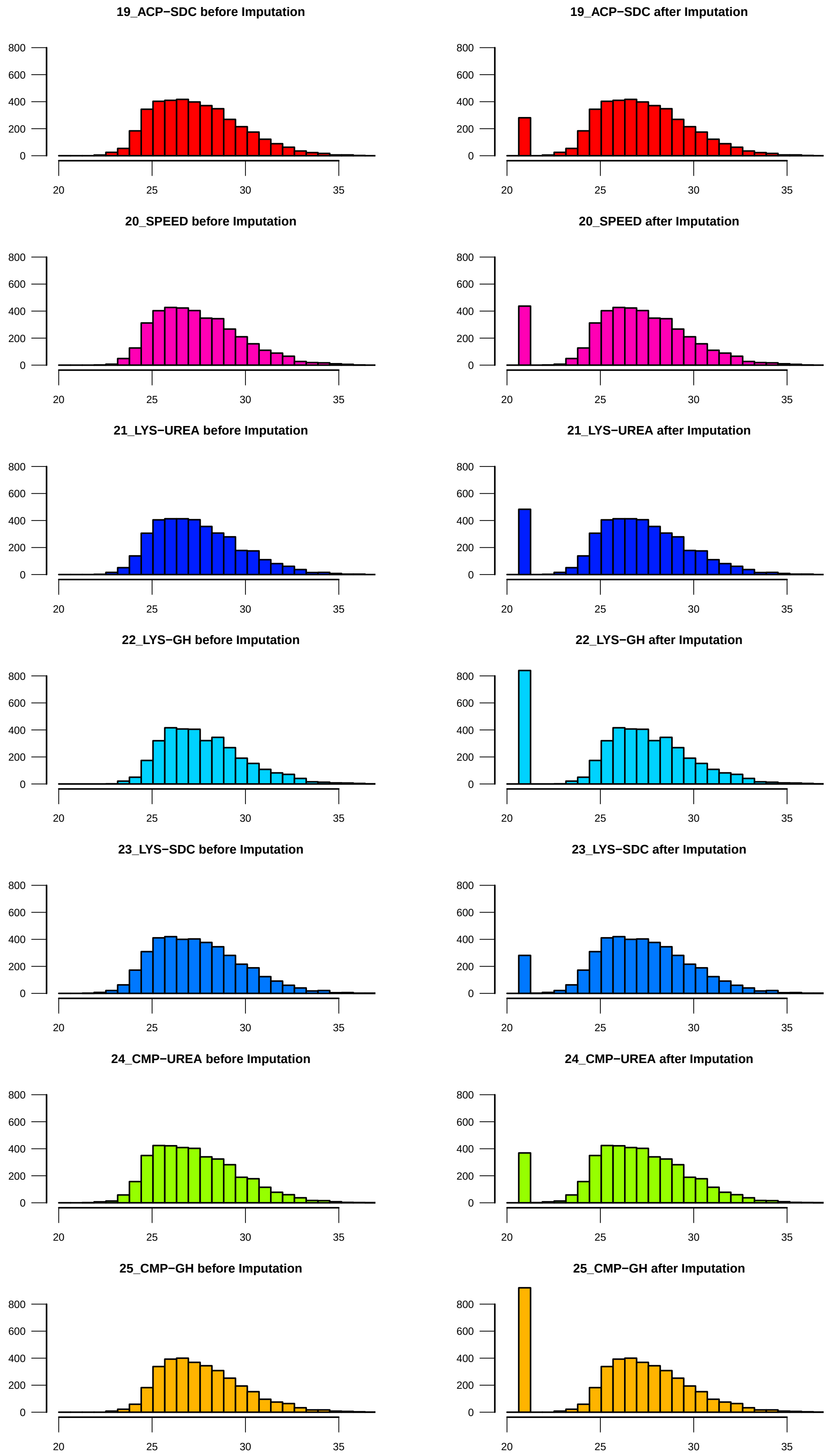
19_ACP−SDC before Imputation
19_ACP−SDC after Imputation
20_SPEED before Imputation
20_SPEED after Imputation
21_LYS−UREA before Imputation
21_LYS−UREA after Imputation
22_LYS−GH before Imputation
22_LYS−GH after Imputation
23_LYS−SDC before Imputation
23_LYS−SDC after Imputation
24_CMP−UREA before Imputation
24_CMP−UREA after Imputation
25_CMP−GH before Imputation
25_CMP−GH after Imputation
0
200
400
600
800
20
25
30
35

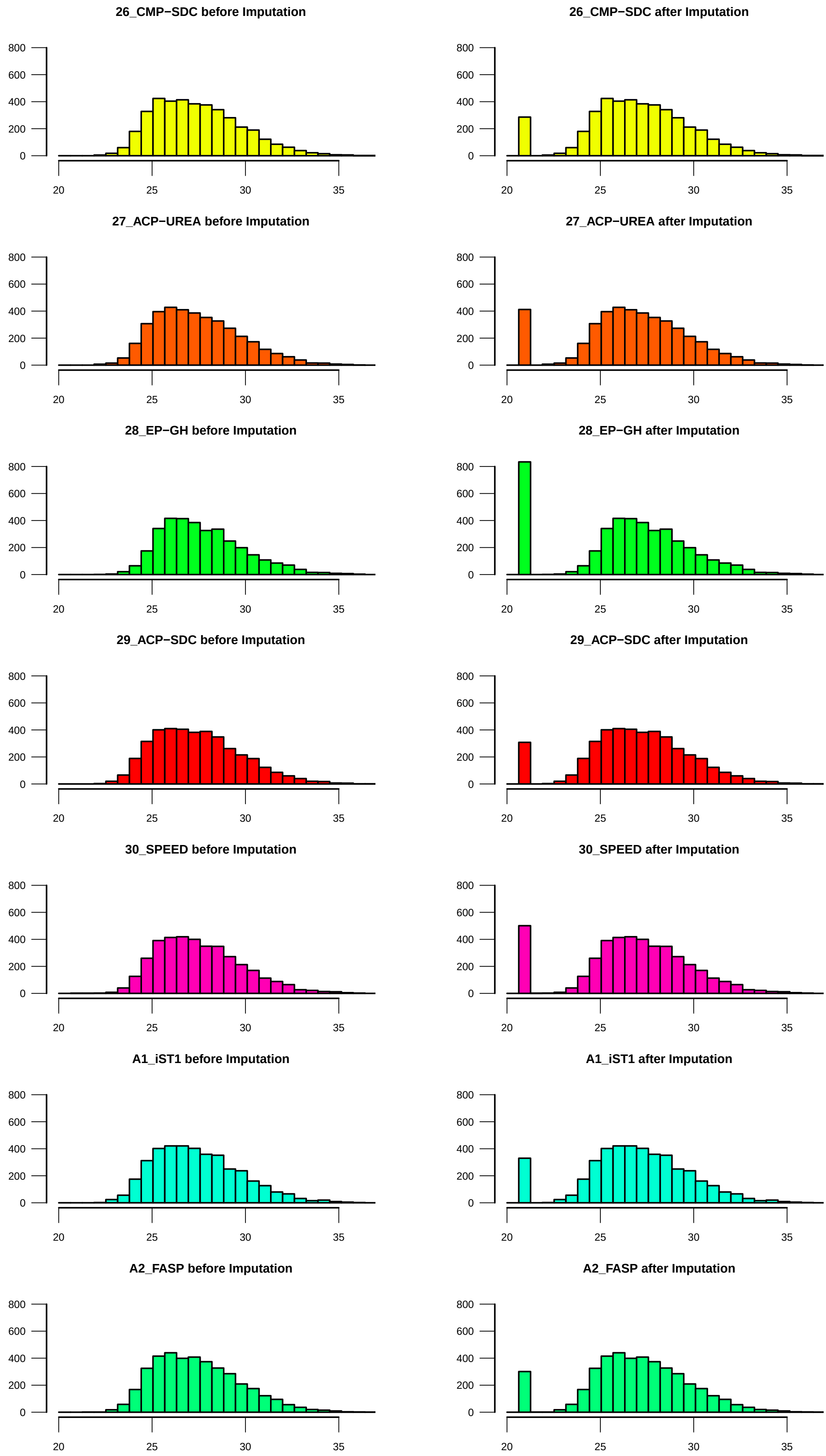
26_CMP−SDC before Imputation
26_CMP−SDC after Imputation
27_ACP−UREA before Imputation
27_ACP−UREA after Imputation
28_EP−GH before Imputation
28_EP−GH after Imputation
29_ACP−SDC before Imputation
29_ACP−SDC after Imputation
30_SPEED before Imputation
30_SPEED after Imputation
A1_iST1 before Imputation
A1_iST1 after Imputation
A2_FASP before Imputation
A2_FASP after Imputation
0
200
400
600
800
20
25
30
35

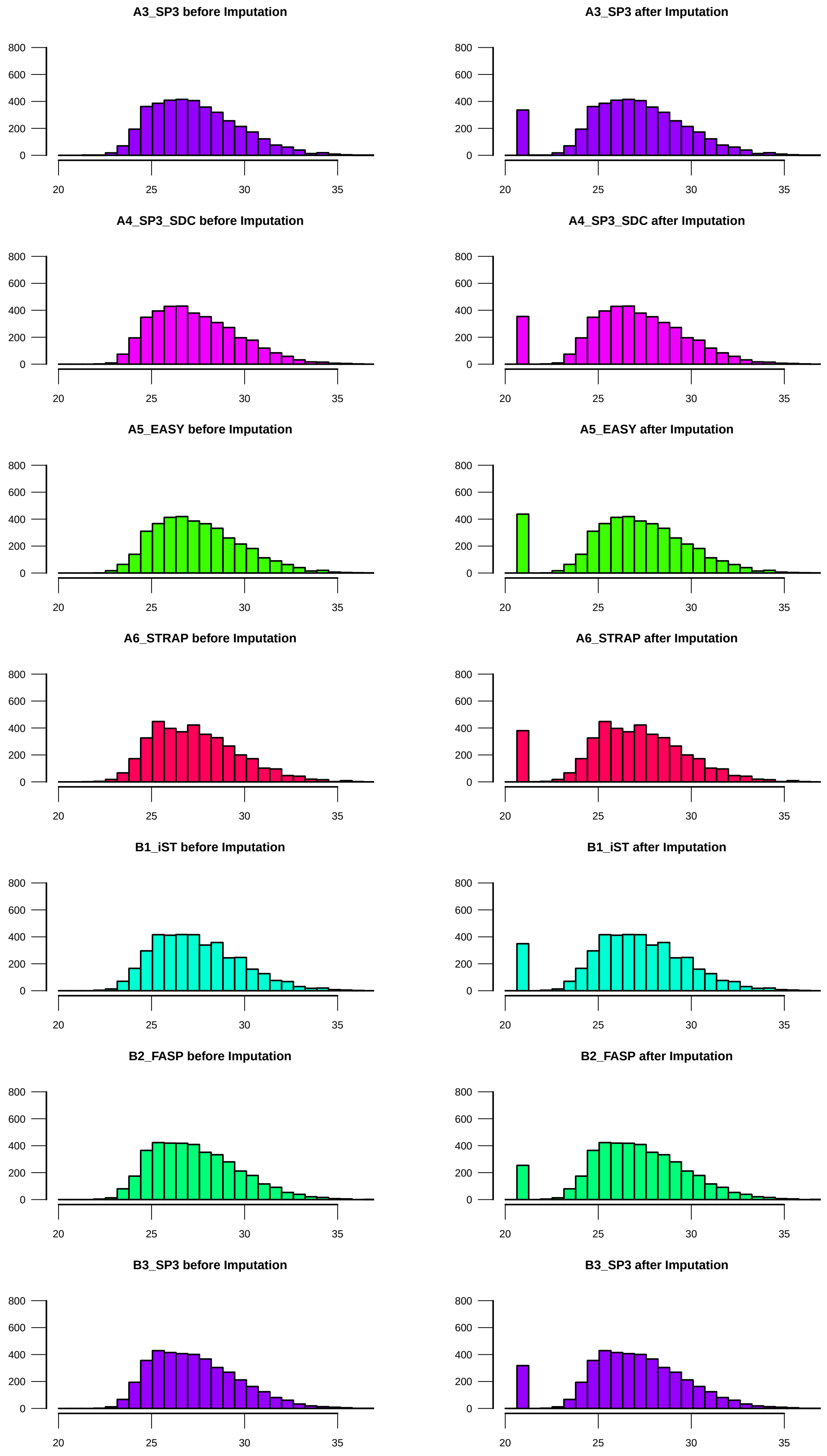
A3_SP3 before Imputation
A3_SP3 after Imputation
A4_SP3_SDC before Imputation
A4_SP3_SDC after Imputation
A5_EASY before Imputation
A5_EASY after Imputation
A6_STRAP before Imputation
A6_STRAP after Imputation
B1_iST before Imputation
B1_iST after Imputation
B2_FASP before Imputation
B2_FASP after Imputation
B3_SP3 before Imputation
B3_SP3 after Imputation

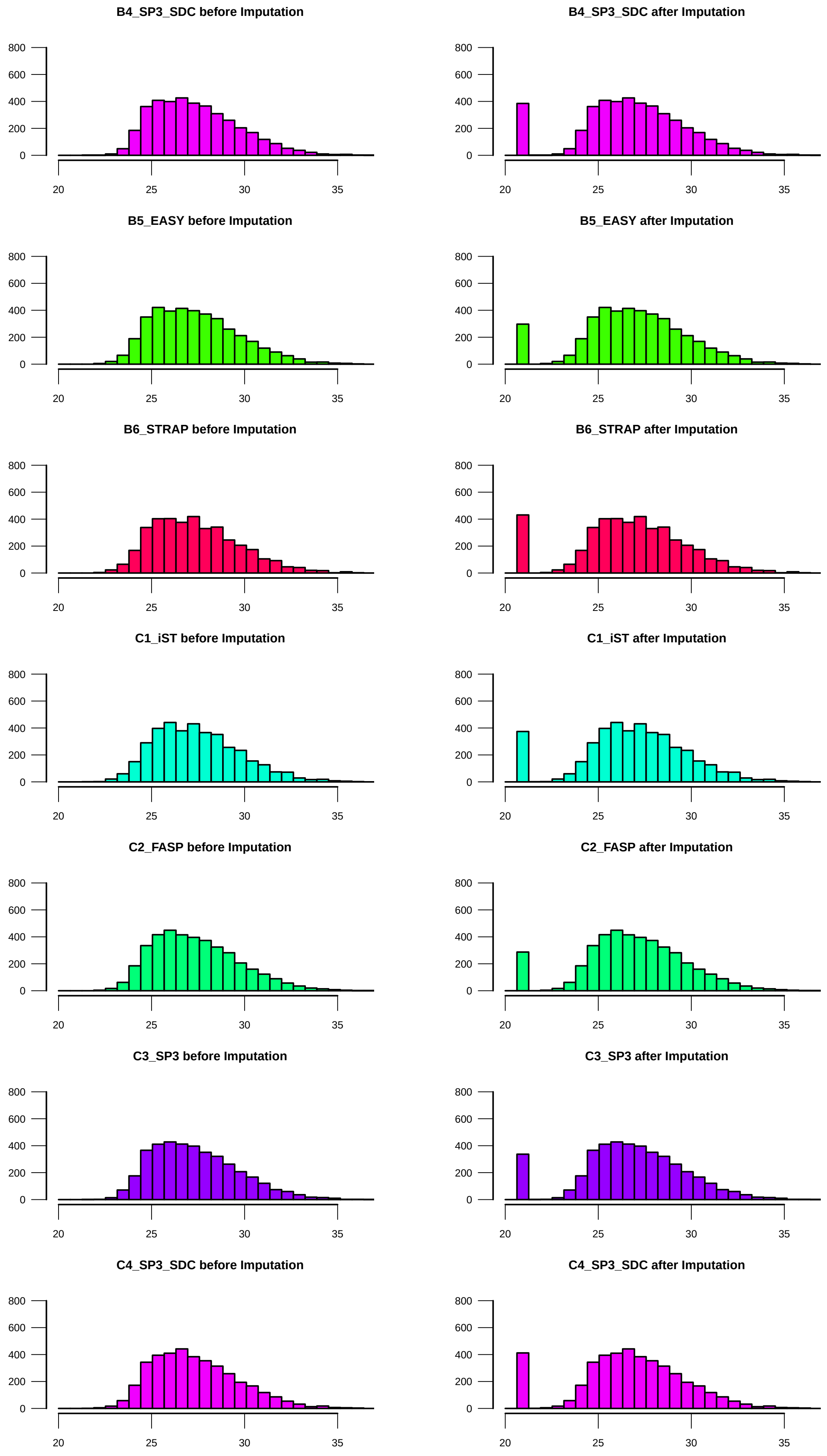
B4_SP3_SDC before Imputation
B4_SP3_SDC after Imputation
B5_EASY before Imputation
B5_EASY after Imputation
B6_STRAP before Imputation
B6_STRAP after Imputation
C1_iST before Imputation
C1_iST after Imputation
C2_FASP before Imputation
C2_FASP after Imputation
C3_SP3 before Imputation
C3_SP3 after Imputation
C4_SP3_SDC before Imputation
C4_SP3_SDC after Imputation
0 200 400 600 800
20 25 30 35

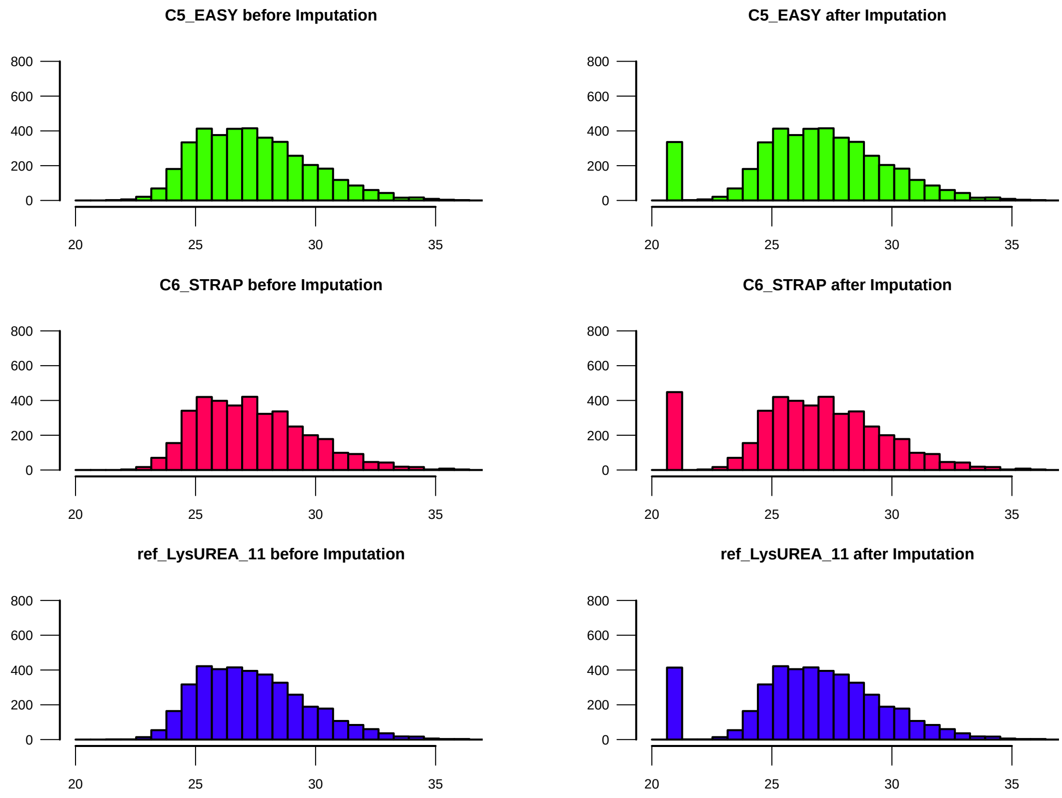

6 Visualization after Imputation

The following visualizations are based on log2 LFQ-intensities of the remaining proteins (rows), with missing data being already imputed.

6.1 Heatmap after Imputation

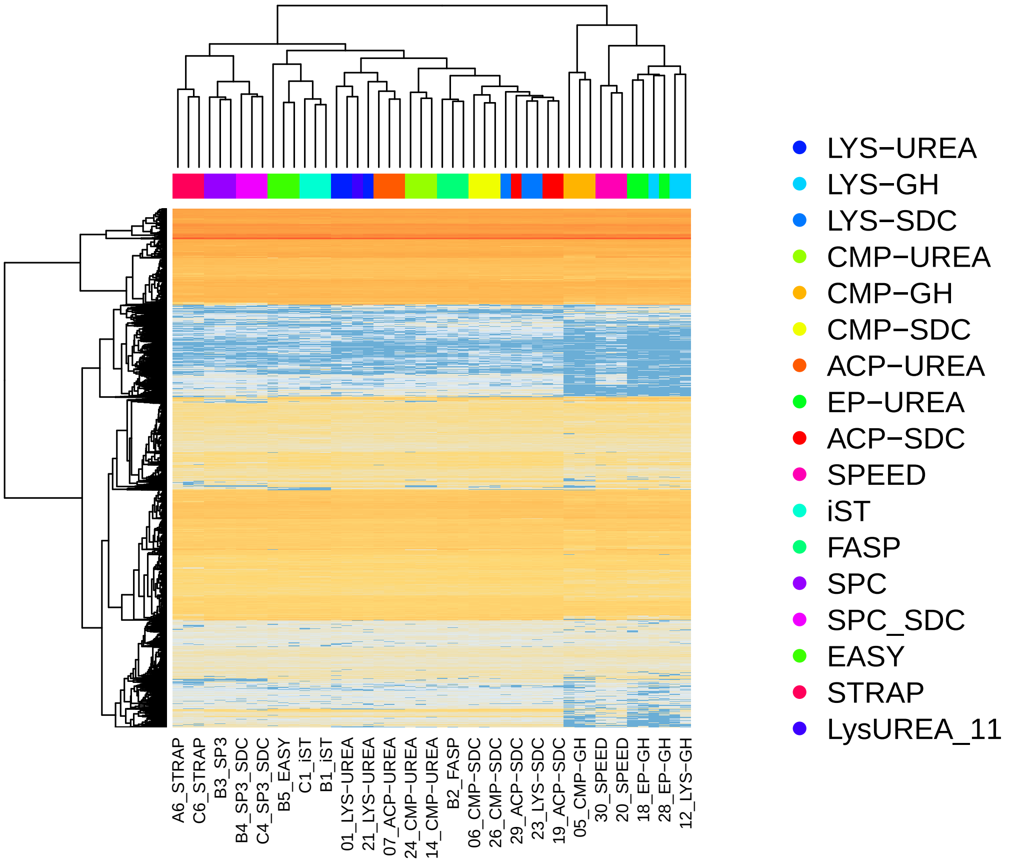

6.2 PCA after Imputation

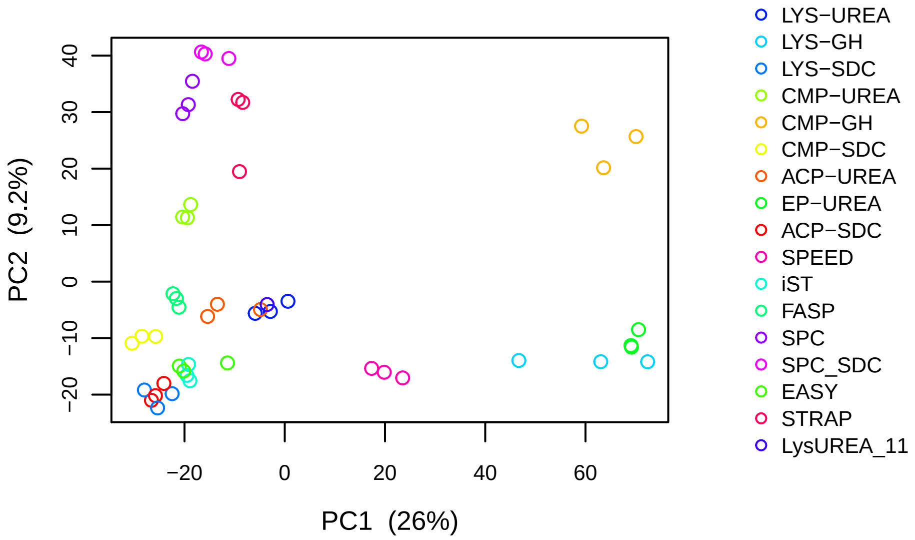

7 Statistical Pairwise Comparison of Groups

7.1 Overview

In this section, Cassiopeia does statistical comparisons of groups using the LIMMA (Linear Models for Microarray Data) package from the R Bioconductor repository. Similar to the classical t-test, LIMMA tests for the equality of LFQ intensity means in two different groups for each protein of proteinGroups.txt (barring those proteins that were removed during filtering). The number of group comparisons in this report are

```
## 0 (out of 136 possible distinct pairwise comparisons)
```

and the groups that are to be compared will be:

```
## NULL
```

The order in which the distinct pairwise comparisons will be reported corresponds to the row-wise order in which they are listed above.

7.2 Results

8 Exploratory Cluster Analysis with k-Means

8.1 Optimal k

```
print(infer_optimal_number_of_clusters)

## [1] TRUE
```

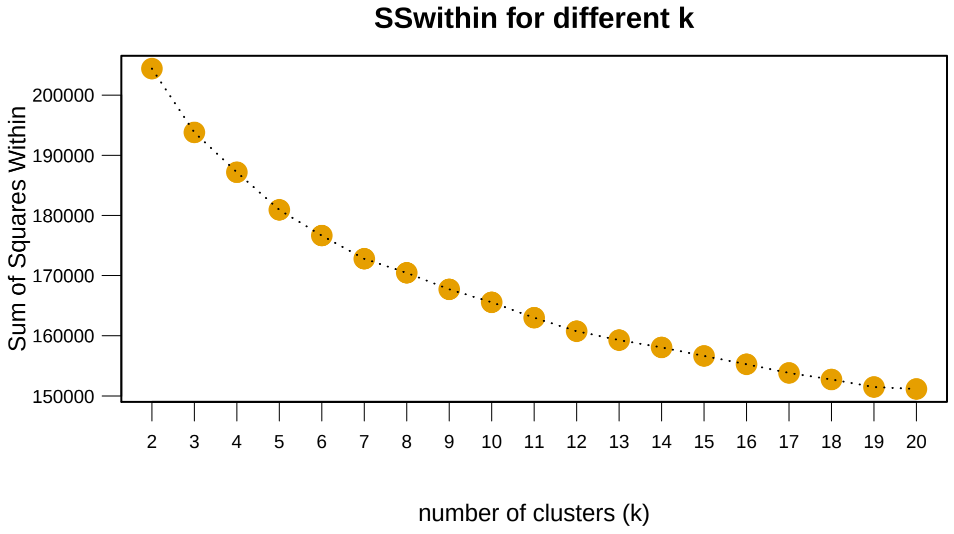

```
## Choose k where:
## The reduction in Sum of Squares Within becomes negligible.
```

8.2 The k Cluster Centers

```
print(number_of_clusters)

## [1] 9

print(export_clusters)

## [1] TRUE
```

Note that before k-Means Clustering, the mean intensity of each protein group (row) is shifted towards a common universal mean, resulting in equal central tendencies for all protein groups (rows). This way, protein groups with similar expression patterns will fall into the same cluster, regardless of differences in absolute expression levels.

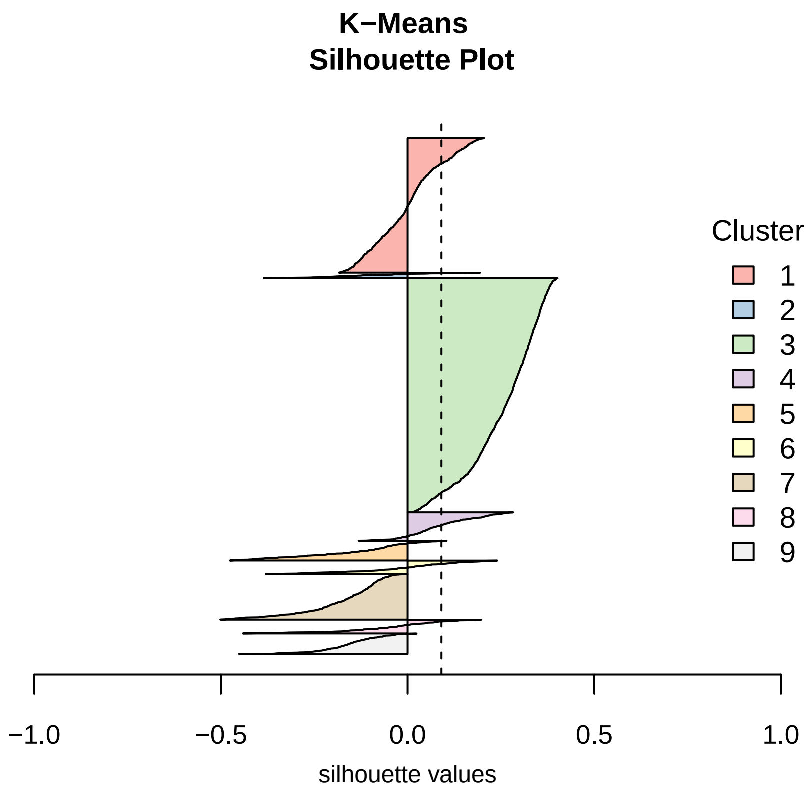


```
## The mean of all silhouette values for this clustering is 0.09
## Note: points with high silhouette values are clustered well
```

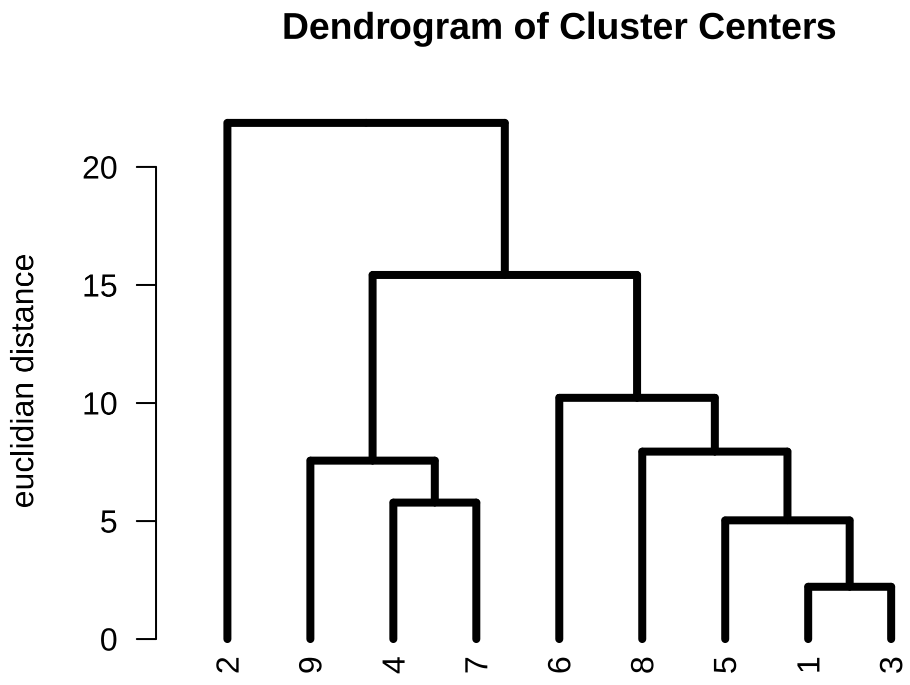


```
## This dendrogram shows an agglomerative clustering of the k-means cluster centers.
## Distances are ultrametric.
```

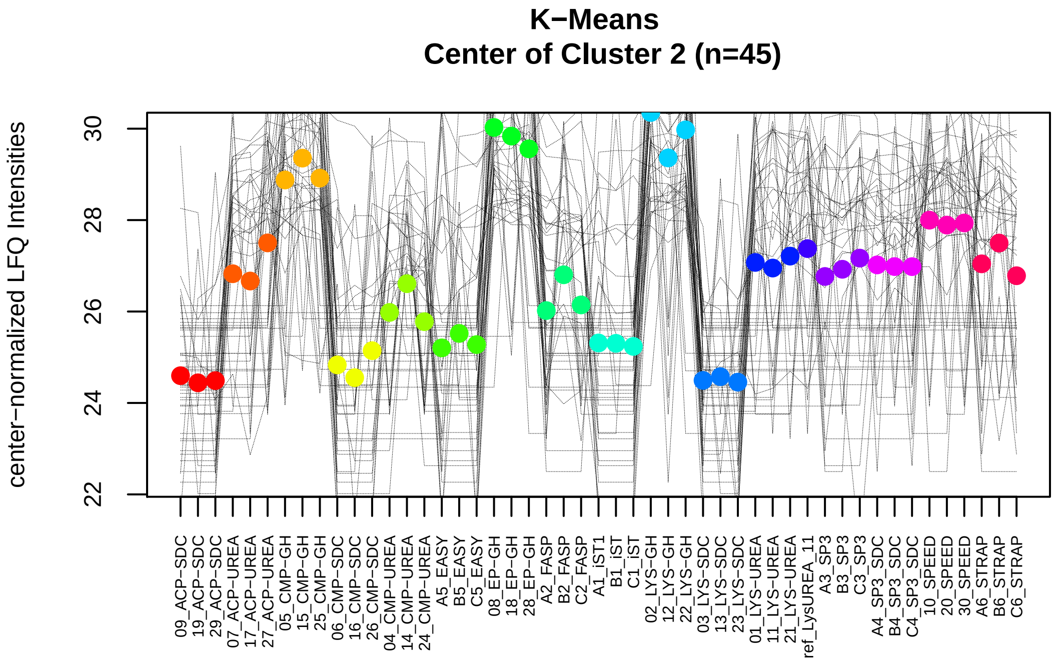

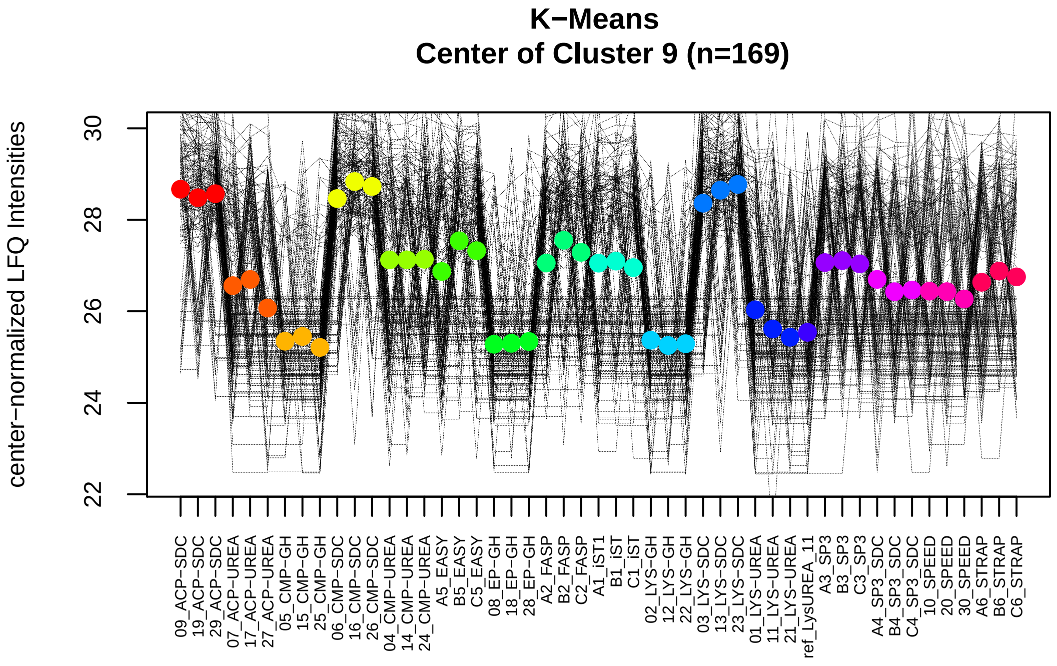
K−Means
Center of Cluster 9 (n=169)
center−normalized LFQ Intensities
22
24
26
28
30
09_ACP−SDC
19_ACP−SDC
29_ACP−SDC
07_ACP−UREA
17_ACP−UREA
27_ACP−UREA
05_CMP−GH
15_CMP−GH
25_CMP−GH
06_CMP−SDC
16_CMP−SDC
26_CMP−SDC
04_CMP−UREA
14_CMP−UREA
24_CMP−UREA
A5_EASY
B5_EASY
C5_EASY
08_EP−GH
18_EP−GH
28_EP−GH
A2_FASP
B2_FASP
C2_FASP
A1_iST1
B1_iST
C1_iST
02_LYS−GH
12_LYS−GH
22_LYS−GH
03_LYS−SDC
13_LYS−SDC
23_LYS−SDC
01_LYS−UREA
11_LYS−UREA
21_LYS−UREA
ref_LysUREA_11
A3_SP3
B3_SP3
C3_SP3
A4_SP3_SDC
B4_SP3_SDC
C4_SP3_SDC
10_SPEED
20_SPEED
30_SPEED
A6_STRAP
B6_STRAP
C6_STRAP

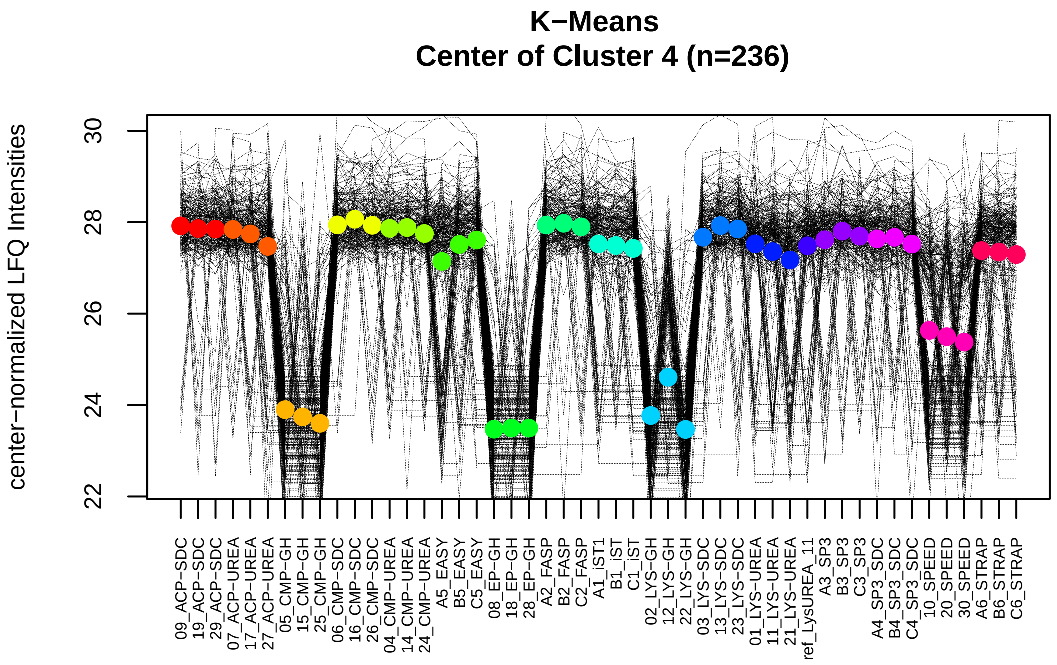
K−Means
Center of Cluster 4 (n=236)
center−normalized LFQ Intensities
22
24
26
28
30
09_ACP−SDC
19_ACP−SDC
29_ACP−SDC
07_ACP−UREA
17_ACP−UREA
27_ACP−UREA
05_CMP−GH
15_CMP−GH
25_CMP−GH
06_CMP−SDC
16_CMP−SDC
26_CMP−SDC
04_CMP−UREA
14_CMP−UREA
24_CMP−UREA
A5_EASY
B5_EASY
C5_EASY
08_EP−GH
18_EP−GH
28_EP−GH
A2_FASP
B2_FASP
C2_FASP
A1_iST1
B1_iST
C1_iST
02_LYS−GH
12_LYS−GH
22_LYS−GH
03_LYS−SDC
13_LYS−SDC
23_LYS−SDC
01_LYS−UREA
11_LYS−UREA
21_LYS−UREA
ref_LysUREA_11
A3_SP3
B3_SP3
C3_SP3
A4_SP3_SDC
B4_SP3_SDC
C4_SP3_SDC
10_SPEED
20_SPEED
30_SPEED
A6_STRAP
B6_STRAP
C6_STRAP

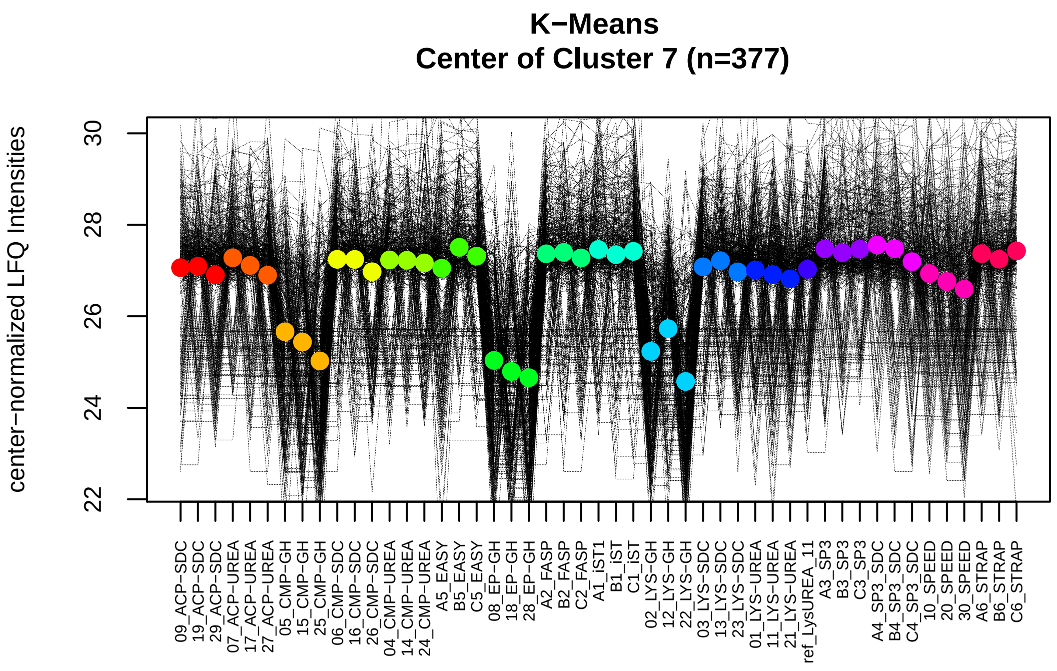
K−Means
Center of Cluster 7 (n=377)
center−normalized LFQ Intensities
22
24
26
28
30
09_ACP−SDC
19_ACP−SDC
29_ACP−SDC
07_ACP−UREA
17_ACP−UREA
27_ACP−UREA
05_CMP−GH
15_CMP−GH
25_CMP−GH
06_CMP−SDC
16_CMP−SDC
26_CMP−SDC
04_CMP−UREA
14_CMP−UREA
24_CMP−UREA
A5_EASY
B5_EASY
C5_EASY
08_EP−GH
18_EP−GH
28_EP−GH
A2_FASP
B2_FASP
C2_FASP
A1_iST1
B1_iST
C1_iST
02_LYS−GH
12_LYS−GH
22_LYS−GH
03_LYS−SDC
13_LYS−SDC
23_LYS−SDC
01_LYS−UREA
11_LYS−UREA
21_LYS−UREA
ref_LysUREA_11
A3_SP3
B3_SP3
C3_SP3
A4_SP3_SDC
B4_SP3_SDC
C4_SP3_SDC
10_SPEED
20_SPEED
30_SPEED
A6_STRAP
B6_STRAP
C6_STRAP

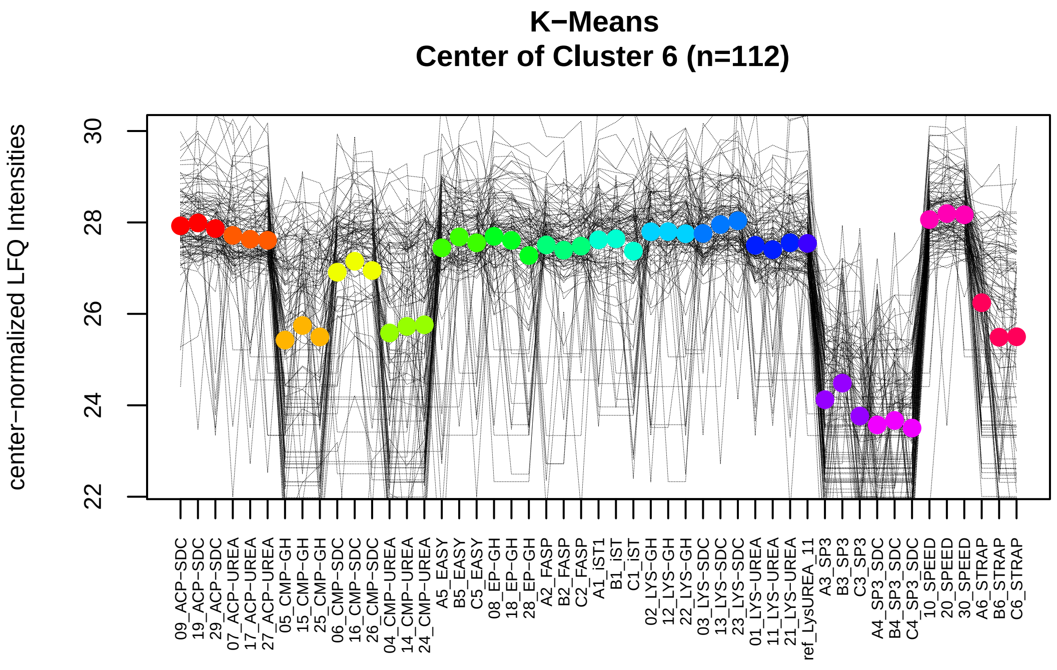
K−Means
Center of Cluster 6 (n=112)
center−normalized LFQ Intensities
22
24
26
28
30
09_ACP−SDC
19_ACP−SDC
29_ACP−SDC
07_ACP−UREA
17_ACP−UREA
27_ACP−UREA
05_CMP−GH
15_CMP−GH
25_CMP−GH
06_CMP−SDC
16_CMP−SDC
26_CMP−SDC
04_CMP−UREA
14_CMP−UREA
24_CMP−UREA
A5_EASY
B5_EASY
C5_EASY
08_EP−GH
18_EP−GH
28_EP−GH
A2_FASP
B2_FASP
C2_FASP
A1_iST1
B1_iST
C1_iST
02_LYS−GH
12_LYS−GH
22_LYS−GH
03_LYS−SDC
13_LYS−SDC
23_LYS−SDC
01_LYS−UREA
11_LYS−UREA
21_LYS−UREA
ref_LysUREA_11
A3_SP3
B3_SP3
C3_SP3
A4_SP3_SDC
B4_SP3_SDC
C4_SP3_SDC
10_SPEED
20_SPEED
30_SPEED
A6_STRAP
B6_STRAP
C6_STRAP

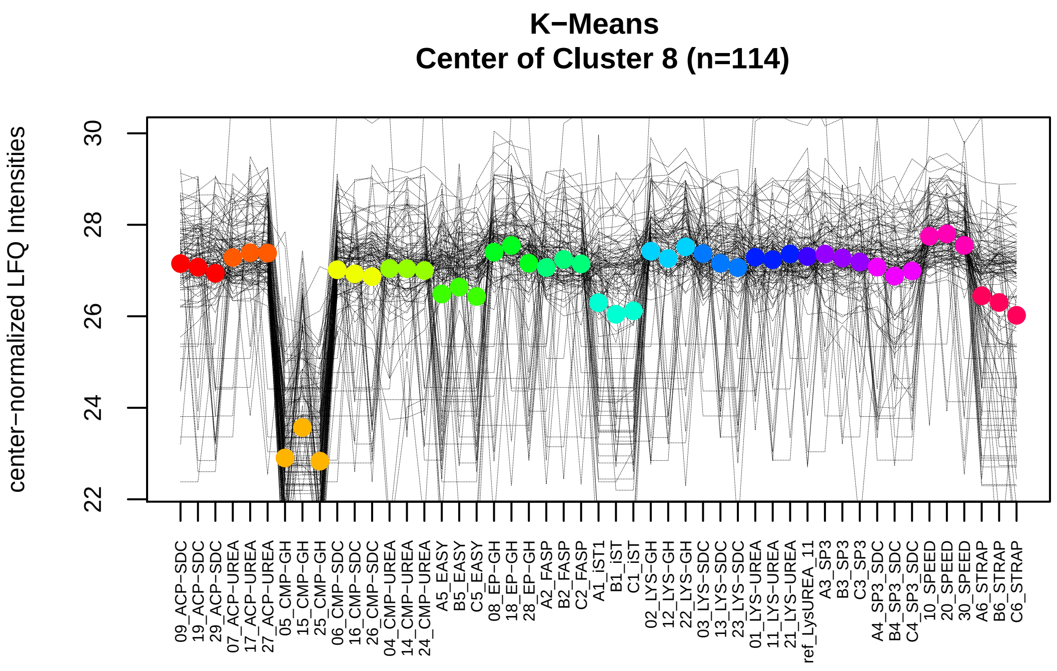
K−Means
Center of Cluster 8 (n=114)
center−normalized LFQ Intensities
22
24
26
28
30
09_ACP-SDC
19_ACP-SDC
29_ACP-SDC
07_ACP-UREA
17_ACP-UREA
27_ACP-UREA
05_CMP-GH
15_CMP-GH
25_CMP-GH
06_CMP-SDC
16_CMP-SDC
26_CMP-SDC
04_CMP-UREA
14_CMP-UREA
24_CMP-UREA
A5_EASY
B5_EASY
C5_EASY
08_EP-GH
18_EP-GH
28_EP-GH
A2_FASP
B2_FASP
C2_FASP
A1_iST1
B1_iST
C1_iST
02_LYS-GH
12_LYS-GH
22_LYS-GH
03_LYS-SDC
13_LYS-SDC
23_LYS-SDC
01_LYS-UREA
11_LYS-UREA
21_LYS-UREA
ref_LysUREA_11
A3_SP3
B3_SP3
C3_SP3
A4_SP3_SDC
B4_SP3_SDC
C4_SP3_SDC
10_SPEED
20_SPEED
30_SPEED
A6_STRAP
B6_STRAP
C6_STRAP

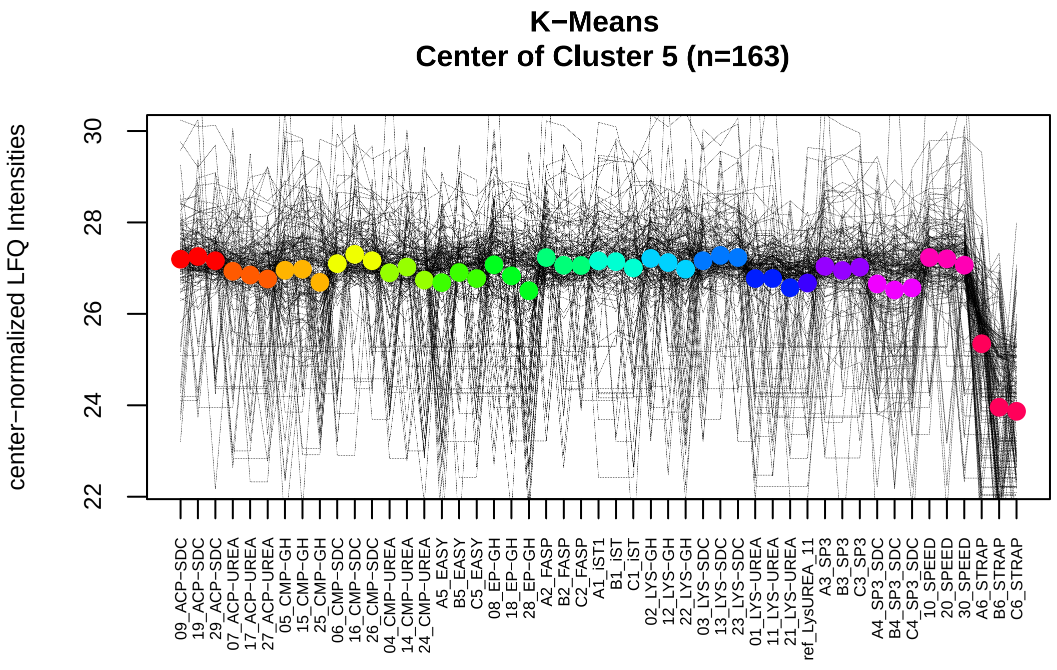
K−Means
Center of Cluster 5 (n=163)
center−normalized LFQ Intensities
22
24
26
28
30
09_ACP-SDC
19_ACP-SDC
29_ACP-SDC
07_ACP-UREA
17_ACP-UREA
27_ACP-UREA
05_CMP-GH
15_CMP-GH
25_CMP-GH
06_CMP-SDC
16_CMP-SDC
26_CMP-SDC
04_CMP-UREA
14_CMP-UREA
24_CMP-UREA
A5_EASY
B5_EASY
C5_EASY
08_EP-GH
18_EP-GH
28_EP-GH
A2_FASP
B2_FASP
C2_FASP
A1_iST1
B1_iST
C1_iST
02_LYS-GH
12_LYS-GH
22_LYS-GH
03_LYS-SDC
13_LYS-SDC
23_LYS-SDC
01_LYS-UREA
11_LYS-UREA
21_LYS-UREA
ref_LysUREA_11
A3_SP3
B3_SP3
C3_SP3
A4_SP3_SDC
B4_SP3_SDC
C4_SP3_SDC
10_SPEED
20_SPEED
30_SPEED
A6_STRAP
B6_STRAP
C6_STRAP

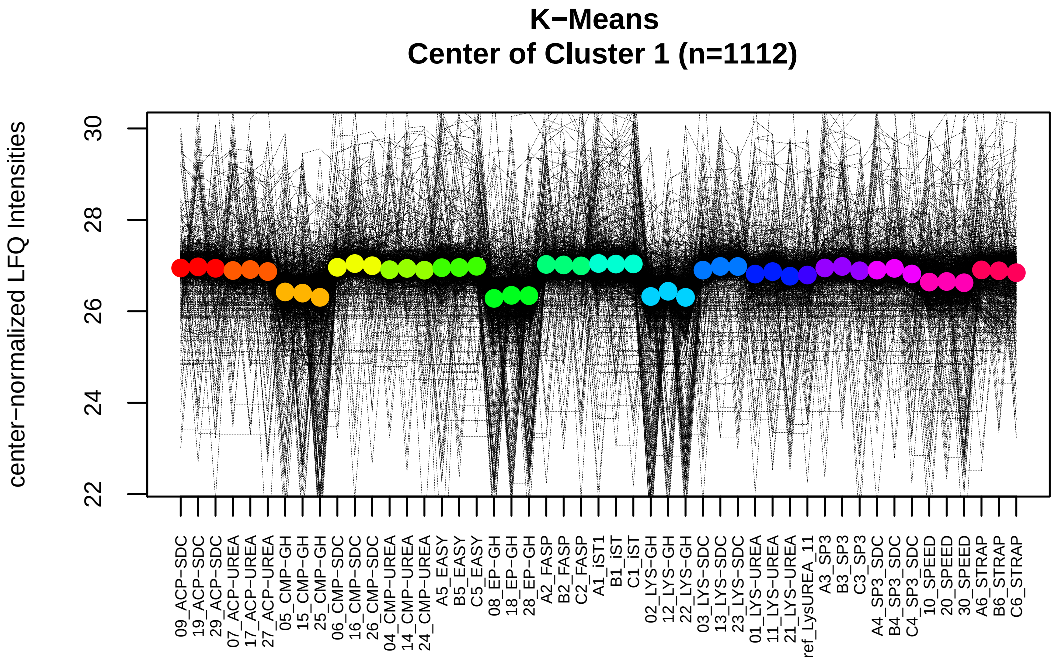

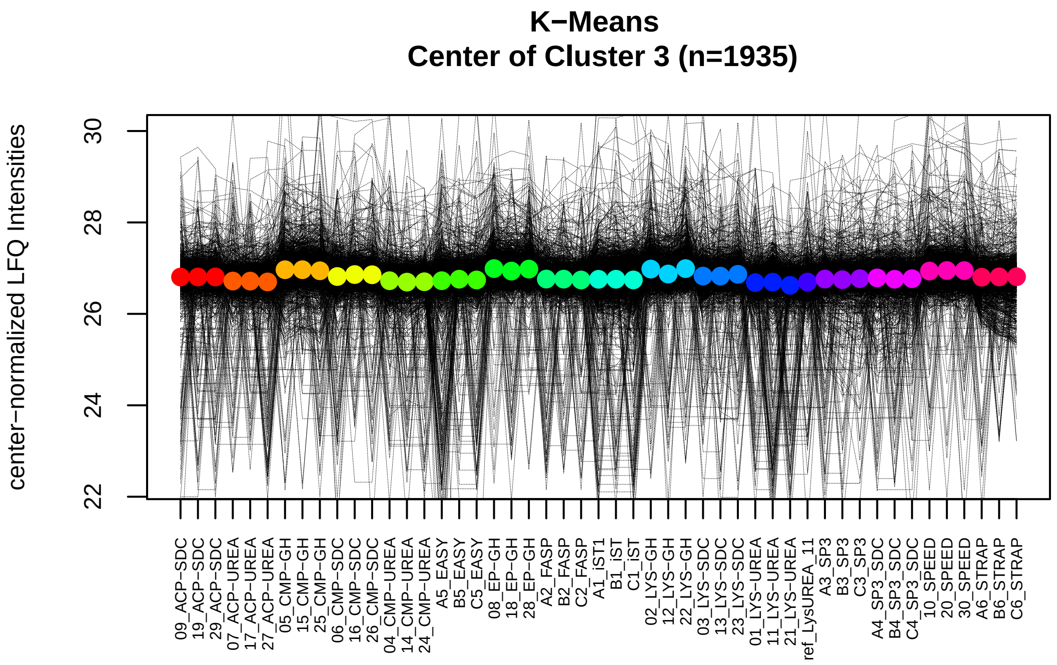

9 Matrix Export

```
print(export_matrix)

## [1] TRUE

print(export_amica)

## [1] FALSE
```

```
## The final data matrix was exported as a tab-delimited text file called:
## Matrix_Export_proteinGroups_gina (2020_11_13 19_25_46 UTC).txt
## The number of rows and columns of this file are:
## 5257 rows, and 522 columns
```
