# Supplementary material for: In Search of a Universal Method: A Comparative Survey of Bottom-Up Proteomics Sample Preparation Methods
Source: J Proteome Res. 2022 Aug 25;21(10):2397–411. doi: 10.1021/acs.jproteome.2c00265 (PMC9552232; doi:10.1021/acs.jproteome.2c00265)
Supplement: Supplementary file 5 — pr2c00265_si_005.zip [file pr2c00265_si_005.zip › Supplemental_Material_Scripts/PTXQC_Output/report_v1.0.5_combined.html]

ProTeomiX (PTX) Quality Control (QC) Report


# ProTeomiX (PTX) Quality Control (QC) Report

- 1 Overview
  - 1.1 HeatMap
  - 1.2 Name Mapping
  - 1.3 Metrics
    - 1.3.1 PAR: parameters
    - 1.3.2 PG: PCA of ‘raw intensity’
    - 1.3.3 PG: PCA of ‘lfq intensity’
    - 1.3.4 EVD: Top5 Contaminants per Raw file
    - 1.3.5 EVD: peptide intensity distribution
    - 1.3.6 PG: intensity distribution
    - 1.3.7 PG: LFQ intensity distribution
    - 1.3.8 MSMS: Missed cleavages per Raw file
    - 1.3.9 EVD: charge distribution
    - 1.3.10 PG: Contaminant per condition
    - 1.3.11 MSMSscans: TopN
    - 1.3.12 MSMSscans: TopN over RT
    - 1.3.13 EVD: IDs over RT
    - 1.3.14 EVD: Peak width over RT
    - 1.3.15 EVD: MBR - alignment
    - 1.3.16 EVD: MBR - ID Transfer
    - 1.3.17 [experimental] EVD: Clustering Tree of Raw files
    - 1.3.18 MSMSscans: Ion Injection Time over RT
    - 1.3.19 [experimental] MSMSscans: MS/MS intensity
    - 1.3.20 EVD: Oversampling (MS/MS counts per 3D-peak)
    - 1.3.21 EVD: Uncalibrated mass error
    - 1.3.22 EVD: Calibrated mass error
    - 1.3.23 MSMS: Fragment mass errors per Raw file
    - 1.3.24 SM: MS/MS identified per Raw file
    - 1.3.25 MSMSscans: TopN % identified over N
    - 1.3.26 [experimental] EVD: Non-Missing Peptides
    - 1.3.27 [experimental] EVD: Non-missing by set
    - 1.3.28 [experimental] EVD: Imputed Peptide Intensity Distribution of Missing Values
    - 1.3.29 EVD: Peptide ID count
    - 1.3.30 EVD: ProteinGroups count
    - 1.3.31 EVD: UpSet distinct

# 1 Overview

**Quick guide**

- navigate using the ‘Table of Content’ above or just scroll down
- collapse sections by just clicking their name
- click on the “↓ Show Help” symbol to see a description for each metric and its scoring
- edit the config file:///X:/Gina/combined/txt/report\_v1.0.5.yaml to
  - set the report format (HTML, plainPDF, or both)
  - disable metrics
  - customize the order of metrics
  - set individual target thresholds
- edit file:///X:/Gina/combined/txt/report\_v1.0.5\_filename\_sort.txt to customize
  - the order of Raw files in plots and heatmap
  - abbreviations of Raw file names
- edit the Html template file:///X:/\_PTXQC/QC-dragdrop/\_internal/R-3.1.0/library/PTXQC/./reportTemplate/PTXQC\_report\_template.Rmd to
  - alter the look and feel of this report (e.g. colors, font and image sizes, help texts, etc.)
- report bugs and file requests via the GitHub issue tracker
- full PTXQC manual available here

## 1.1 HeatMap

## 1.2 Name Mapping

| Mapping of Raw files to their short names Mapping source: automatic (automatic shortening of names was not sufficient - see ‘best effort’) | | |
| --- | --- | --- |
| from | to | best.effort |
| 20200520\_QExHFX1\_RSLC1\_Anrather\_Hartl\_MFPL\_A1\_iST1\_iRT\_500ng | file 01 | ..0\_Q..L\_A1\_iST1\_i..ng |
| 20200520\_QExHFX1\_RSLC1\_Anrather\_Hartl\_MFPL\_A2\_FASP\_iRT\_500ng | file 02 | ..0\_Q..L\_A2\_FASP\_i..ng |
| 20200520\_QExHFX1\_RSLC1\_Anrather\_Hartl\_MFPL\_A3\_SPC\_iRT\_500ng | file 03 | ..0\_Q..L\_A3\_SPC\_i..ng |
| 20200520\_QExHFX1\_RSLC1\_Anrather\_Hartl\_MFPL\_A4\_SPC\_SDC\_iRT\_500ng | file 04 | ..0\_Q..L\_A4\_SPC\_SDC\_i..ng |
| 20200520\_QExHFX1\_RSLC1\_Anrather\_Hartl\_MFPL\_A5\_EASY\_iRT\_500ng | file 05 | ..0\_Q..L\_A5\_EASY\_i..ng |
| 20200520\_QExHFX1\_RSLC1\_Anrather\_Hartl\_MFPL\_A6\_STRAP\_iRT\_500ng | file 06 | ..0\_Q..L\_A6\_STRAP\_i..ng |
| 20200520\_QExHFX1\_RSLC1\_Anrather\_Hartl\_MFPL\_B1\_iST\_iRT\_500ng | file 07 | ..0\_Q..L\_B1\_iST\_i..ng |
| 20200520\_QExHFX1\_RSLC1\_Anrather\_Hartl\_MFPL\_B2\_FASP\_iRT\_500ng | file 08 | ..0\_Q..L\_B2\_FASP\_i..ng |
| 20200520\_QExHFX1\_RSLC1\_Anrather\_Hartl\_MFPL\_B3\_SPC\_iRT\_500ng | file 09 | ..0\_Q..L\_B3\_SPC\_i..ng |
| 20200520\_QExHFX1\_RSLC1\_Anrather\_Hartl\_MFPL\_B4\_SPC\_SDC\_iRT\_500ng | file 10 | ..0\_Q..L\_B4\_SPC\_SDC\_i..ng |
| 20200520\_QExHFX1\_RSLC1\_Anrather\_Hartl\_MFPL\_B5\_EASY\_iRT\_500ng | file 11 | ..0\_Q..L\_B5\_EASY\_i..ng |
| 20200520\_QExHFX1\_RSLC1\_Anrather\_Hartl\_MFPL\_B6\_STRAP\_iRT\_500ng | file 12 | ..0\_Q..L\_B6\_STRAP\_i..ng |
| 20200520\_QExHFX1\_RSLC1\_Anrather\_Hartl\_MFPL\_C1\_iST\_iRT\_500ng | file 13 | ..0\_Q..L\_C1\_iST\_i..ng |
| 20200520\_QExHFX1\_RSLC1\_Anrather\_Hartl\_MFPL\_C2\_FASP\_iRT\_500ng | file 14 | ..0\_Q..L\_C2\_FASP\_i..ng |
| 20200520\_QExHFX1\_RSLC1\_Anrather\_Hartl\_MFPL\_C3\_SPC\_iRT\_500ng | file 15 | ..0\_Q..L\_C3\_SPC\_i..ng |
| 20200520\_QExHFX1\_RSLC1\_Anrather\_Hartl\_MFPL\_C4\_SPC\_SDC\_iRT\_500ng | file 16 | ..0\_Q..L\_C4\_SPC\_SDC\_i..ng |
| 20200520\_QExHFX1\_RSLC1\_Anrather\_Hartl\_MFPL\_C5\_EASY\_iRT\_500ng | file 17 | ..0\_Q..L\_C5\_EASY\_i..ng |
| 20200520\_QExHFX1\_RSLC1\_Anrather\_Hartl\_MFPL\_C6\_STRAP\_iRT\_500ng | file 18 | ..0\_Q..L\_C6\_STRAP\_i..ng |
| 20200520\_QExHFX1\_RSLC1\_Anrather\_Hartl\_MFPL\_ref\_LysUREA\_11\_iRT\_500ng | file 19 | ..0\_Q..L\_ref\_LysUREA\_11\_i..ng |
| 20200520\_QExHFX1\_RSLC1\_Anrather\_Hartl\_MFPL\_01\_LYS-UREA\_iRT\_500ng | file 20 | ..0\_Q..L\_01\_LYS-UREA\_i..ng |
| 20200520\_QExHFX1\_RSLC1\_Anrather\_Hartl\_MFPL\_02\_LYS-GH\_iRT\_500ng | file 21 | ..0\_Q..L\_02\_LYS-GH\_i..ng |
| 20200520\_QExHFX1\_RSLC1\_Anrather\_Hartl\_MFPL\_03\_LYS-SDC\_iRT\_500ng | file 22 | ..0\_Q..L\_03\_LYS-SDC\_i..ng |
| 20200520\_QExHFX1\_RSLC1\_Anrather\_Hartl\_MFPL\_04\_CMP-UREA\_iRT\_500ng | file 23 | ..0\_Q..L\_04\_CMP-UREA\_i..ng |
| 20200520\_QExHFX1\_RSLC1\_Anrather\_Hartl\_MFPL\_05\_CMP-GH\_iRT\_500ng | file 24 | ..0\_Q..L\_05\_CMP-GH\_i..ng |
| 20200520\_QExHFX1\_RSLC1\_Anrather\_Hartl\_MFPL\_06\_CMP-SDC\_iRT\_500ng | file 25 | ..0\_Q..L\_06\_CMP-SDC\_i..ng |
| 20200520\_QExHFX1\_RSLC1\_Anrather\_Hartl\_MFPL\_07\_ACP-UREA\_iRT\_500ng | file 26 | ..0\_Q..L\_07\_ACP-UREA\_i..ng |
| 20200520\_QExHFX1\_RSLC1\_Anrather\_Hartl\_MFPL\_08\_EP-UREA\_iRT\_500ng | file 27 | ..0\_Q..L\_08\_EP-UREA\_i..ng |
| 20200520\_QExHFX1\_RSLC1\_Anrather\_Hartl\_MFPL\_09\_ACP-SDC\_iRT\_500ng | file 28 | ..0\_Q..L\_09\_ACP-SDC\_i..ng |
| 20200520\_QExHFX1\_RSLC1\_Anrather\_Hartl\_MFPL\_10\_SPEED\_iRT\_500ng | file 29 | ..0\_Q..L\_10\_SPEED\_i..ng |
| 20200525\_QExHFX1\_RSLC1\_Anrather\_Hartl\_MFPL\_11\_LYS-UREA\_iRT\_500ng | file 30 | ..5\_Q..L\_11\_LYS-UREA\_i..ng |
| 20200525\_QExHFX1\_RSLC1\_Anrather\_Hartl\_MFPL\_12\_LYS-GH\_iRT\_500ng | file 31 | ..5\_Q..L\_12\_LYS-GH\_i..ng |
| 20200525\_QExHFX1\_RSLC1\_Anrather\_Hartl\_MFPL\_13\_LYS-SDC\_iRT\_500ng | file 32 | ..5\_Q..L\_13\_LYS-SDC\_i..ng |
| 20200525\_QExHFX1\_RSLC1\_Anrather\_Hartl\_MFPL\_14\_CMP-UREA\_iRT\_500ng | file 33 | ..5\_Q..L\_14\_CMP-UREA\_i..ng |
| 20200525\_QExHFX1\_RSLC1\_Anrather\_Hartl\_MFPL\_15\_CMP-GH\_iRT\_500ng | file 34 | ..5\_Q..L\_15\_CMP-GH\_i..ng |
| 20200525\_QExHFX1\_RSLC1\_Anrather\_Hartl\_MFPL\_16\_CMP-SDC\_iRT\_500ng | file 35 | ..5\_Q..L\_16\_CMP-SDC\_i..ng |
| 20200525\_QExHFX1\_RSLC1\_Anrather\_Hartl\_MFPL\_17\_ACP-UREA\_iRT\_500ng | file 36 | ..5\_Q..L\_17\_ACP-UREA\_i..ng |
| 20200525\_QExHFX1\_RSLC1\_Anrather\_Hartl\_MFPL\_18\_EP-UREA\_iRT\_500ng | file 37 | ..5\_Q..L\_18\_EP-UREA\_i..ng |
| 20200525\_QExHFX1\_RSLC1\_Anrather\_Hartl\_MFPL\_19\_ACP-SDC\_iRT\_500ng | file 38 | ..5\_Q..L\_19\_ACP-SDC\_i..ng |
| 20200525\_QExHFX1\_RSLC1\_Anrather\_Hartl\_MFPL\_20\_SPEED\_iRT\_500ng | file 39 | ..5\_Q..L\_20\_SPEED\_i..ng |
| 20200525\_QExHFX1\_RSLC1\_Anrather\_Hartl\_MFPL\_21\_LYS-UREA\_iRT\_500ng | file 40 | ..5\_Q..L\_21\_LYS-UREA\_i..ng |
| 20200525\_QExHFX1\_RSLC1\_Anrather\_Hartl\_MFPL\_22\_LYS-GH\_iRT\_500ng | file 41 | ..5\_Q..L\_22\_LYS-GH\_i..ng |
| 20200525\_QExHFX1\_RSLC1\_Anrather\_Hartl\_MFPL\_23\_LYS-SDC\_iRT\_500ng | file 42 | ..5\_Q..L\_23\_LYS-SDC\_i..ng |
| 20200525\_QExHFX1\_RSLC1\_Anrather\_Hartl\_MFPL\_24\_CMP-UREA\_iRT\_500ng | file 43 | ..5\_Q..L\_24\_CMP-UREA\_i..ng |
| 20200525\_QExHFX1\_RSLC1\_Anrather\_Hartl\_MFPL\_25\_CMP-GH\_iRT\_500ng | file 44 | ..5\_Q..L\_25\_CMP-GH\_i..ng |
| 20200525\_QExHFX1\_RSLC1\_Anrather\_Hartl\_MFPL\_26\_CMP-SDC\_iRT\_500ng | file 45 | ..5\_Q..L\_26\_CMP-SDC\_i..ng |
| 20200525\_QExHFX1\_RSLC1\_Anrather\_Hartl\_MFPL\_27\_ACP-UREA\_iRT\_500ng | file 46 | ..5\_Q..L\_27\_ACP-UREA\_i..ng |
| 20200525\_QExHFX1\_RSLC1\_Anrather\_Hartl\_MFPL\_28\_EP-UREA\_iRT\_500ng | file 47 | ..5\_Q..L\_28\_EP-UREA\_i..ng |
| 20200525\_QExHFX1\_RSLC1\_Anrather\_Hartl\_MFPL\_29\_ACP-SDC\_iRT\_500ng | file 48 | ..5\_Q..L\_29\_ACP-SDC\_i..ng |
| 20200525\_QExHFX1\_RSLC1\_Anrather\_Hartl\_MFPL\_30\_SPEED\_iRT\_500ng | file 49 | ..5\_Q..L\_30\_SPEED\_i..ng |

## 1.3 Metrics

### 1.3.1 PAR: parameters

↓ Show Help

MaxQuant parameters, extracted from parameters.txt (abbreviated as ‘PAR’), summarizes the settings used for the MaxQuant analysis. Key parameters are MaxQuant version, Re-quantify, Match-between-runs and mass search tolerances. A list of protein database files is also provided, allowing to track database completeness and database version information (if given in the filename).

| iRT.fasta 2020.01\_UP000005640\_9606\_Homo\_sapiens\_canonical.fasta | | | |
| --- | --- | --- | --- |
| parameter | value | parameter | value |
| Advanced ratios | True | Min. peptide Length | 7 |
| Alignment ion mobility windo.. | 1 | Min. peptide length for unsp.. | 8 |
| Alignment time window [min] | 20 | Min. score for modified pept.. | 40 |
| Calculate peak properties | False | Modifications included in pr.. | Oxidation (M) Acetyl (Protein N-term) |
| Combined folder location |  | MS/MS deisotoping tolerance .. | ppm |
| Da interval. (FTMS) | 100 | MS/MS deisotoping tolerance .. | 0.15 |
| Date of writing | 07/25/2020 07:36:00 | MS/MS deisotoping tolerance .. | Da |
| Decoy mode | revert | MS/MS deisotoping tolerance .. | 0.01 |
| Label min. ratio count | 2 | MS/MS tol. (FTMS) | 20 ppm |
| Machine name | A240-MASS-DEV01 | MS/MS tol. (ITMS) | 0.5 Da |
| Main search max. combination.. | 200 | MS/MS tol. (TOF) | 40 ppm |
| Match ion mobility window [i.. | 0.05 | Peptides used for protein qu.. | Razor |
| Matching time window [min] | 0.7 | Site tables | Oxidation (M)Sites.txt |
| Max mods in site table | 3 | Top MS/MS peaks per Da inter.. | 12 |
| Max. peptide length for unsp.. | 25 | Top MS/MS peaks per Da inter.. | 10 |
| Max. peptide mass [Da] | 4600 | User name | masse |
| Min. delta score for modifie.. | 6 | Variation mode | None |
| Min. delta score for unmodif.. | 0 | Version | 1.6.14.0 |

back to top

### 1.3.2 PG: PCA of ‘raw intensity’

**(excludes contaminants)**

↓ Show Help

Principal components plots of experimental groups (as defined during MaxQuant configuration).

This plot is shown only if more than one experimental group was defined. If LFQ was activated in MaxQuant, an additional PCA plot for LFQ intensities is shown. Similarly, if iTRAQ/TMT reporter intensities are detected.

Since experimental groups and Raw files do not necessarily correspond 1:1, this plot cannot use the abbreviated Raw file names, but instead must rely on automatic shortening of group names.

Heatmap score: none (since data source proteinGroups.txt is not related 1:1 to Raw files)

### 1.3.3 PG: PCA of ‘lfq intensity’

**(excludes contaminants)**

back to top

### 1.3.4 EVD: Top5 Contaminants per Raw file

↓ Show Help

PTXQC will explicitly show the five most abundant external protein contaminants (as detected via MaxQuant’s contaminants FASTA file) by Raw file, and summarize the remaining contaminants as ‘other’. This allows to track down which proteins exactly contaminate your sample. Low contamination is obviously better. The ‘Abundance class’ models the average peptide intensity in each Raw file and is visualized using varying degrees of transparency. It is not unusual to see samples with low sample content to have higher contamination. If you see only one abundance class (‘mid’), this means all your Raw files have roughly the same peptide intensity distribution.

Heatmap score [EVD: Contaminants]: as fraction of summed intensity with 0 = sample full of contaminants; 1 = no contaminants

back to top

### 1.3.5 EVD: peptide intensity distribution

**RSD 1.1% (expected < 5%)**

↓ Show Help

Peptide precursor intensity per Raw file from evidence.txt WITHOUT match-between-runs evidence. Low peptide intensity usually goes hand in hand with low MS/MS identifcation rates and unfavourable signal/noise ratios, which makes signal detection harder. Also instrument acquisition time increases for trapping instruments.

Failing to reach the intensity threshold is usually due to unfavorable column conditions, inadequate column loading or ionization issues. If the study is not a dilution series or pulsed SILAC experiment, we would expect every condition to have about the same median log-intensity (of 223.0). The relative standard deviation (RSD) gives an indication about reproducibility across files and should be below 5%.

Depending on your setup, your target thresholds might vary from PTXQC’s defaults. Change the threshold using the YAML configuration file.

Heatmap score [EVD: Pep Intensity (>23.0)]: Linear scale of the median intensity reaching the threshold, i.e. reaching 221 of 223 gives score 0.25.

back to top

### 1.3.6 PG: intensity distribution

**RSD 1.3% (w/o zero int.; expected < 5%)% [high RSD –> few peptides])**

↓ Show Help

Intensity boxplots by experimental groups. Groups are user-defined during MaxQuant configuration. This plot displays a (customizable) threshold line for the desired mean intensity of proteins. Groups which underperform here, are likely to also suffer from a worse MS/MS id rate and higher contamination due to the lack of total protein loaded/detected. If possible, all groups should show a high and consistent amount of total protein. The height of the bar correlates to the number of proteins with non-zero abundance.

Contaminants are shown as overlayed yellow boxes, whose height corresponds to the number of contaminant proteins. The position of the box gives the intensity distribution of the contaminants.

Heatmap score: none (since data source proteinGroups.txt is not related 1:1 to Raw files)

back to top

### 1.3.7 PG: LFQ intensity distribution

**RSD 0.4% (w/o zero int.; expected < 5%)% [high RSD –> few peptides])**

↓ Show Help

Label-free quantification (LFQ) intensity boxplots by experimental groups. Groups are user-defined during MaxQuant configuration. This plot displays a (customizable) threshold line for the desired mean of LFQ intensity of proteins. Raw files which underperform in *Raw* intensity, are likely to show an *increased* mean here, since only high-abundance proteins are recovered and quantifyable by MaxQuant in this Raw file. The remaining proteins are likely to receive an LFQ value of 0 (i.e. do not contribute to the distribution). The height of the bar correlates to the number of proteins with non-zero abundance.

Contaminants are shown as overlayed yellow boxes, whose height corresponds to the number of contaminant proteins. The position of the box gives the intensity distribution of the contaminants.

Heatmap score: none (since data source proteinGroups.txt is not related 1:1 to Raw files)

back to top

### 1.3.8 MSMS: Missed cleavages per Raw file

**(excludes contaminants)**

↓ Show Help

Under optimal digestion conditions (high enzyme grade etc.), only few missed cleavages (MC) are expected. In general, increased MC counts also increase the number of peptide signals, thus cluttering the available space and potentially provoking overlapping peptide signals, biasing peptide quantification. Thus, low MC counts should be favored. Interestingly, it has been shown recently that incorporation of peptides with missed cleavages does not negatively influence protein quantification (see Chiva, C., Ortega, M., and Sabido, E. Influence of the Digestion Technique, Protease, and Missed Cleavage Peptides in Protein Quantitation. J. Proteome Res. 2014, 13, 3979-86 ). However this is true only if all samples show the same degree of digestion. High missed cleavage values can indicate for example, either a) failed digestion, b) a high (post-digestion) protein contamination, or c) a sample with high amounts of unspecifically degraded peptides which are not digested by trypsin.

If MC>=1 is high (>20%) you should increase the missed cleavages settings in MaxQuant and compare the number of peptides. Usually high MC correlates with bad identification rates, since many spectra cannot be matched to the forward database.

In the rare case that ‘no enzyme’ was specified in MaxQuant, neither scores nor plots are shown.

Heatmap score [MSMS: MC]: the fraction (0% - 100%) of fully cleaved peptides per Raw file

Heatmap score [MSMS: MC Var]: each Raw file is scored for its deviation (score: MedianDist) from the ‘average’ digestion state of the current study.

back to top

### 1.3.9 EVD: charge distribution

↓ Show Help

Charge distribution per Raw file. For typtic digests, peptides of charge 2 (one N-terminal and one at tryptic C-terminal R or K residue) should be dominant. Ionization issues (voltage?), in-source fragmentation, missed cleavages and buffer irregularities can cause a shift (see Bittremieux 2017, DOI: 10.1002/mas.21544 ). The charge distribution should be similar across Raw files. Consistent charge distribution is paramount for comparable 3D-peak intensities across samples.

Heatmap score [EVD: Charge]: Deviation of the charge 2 proportion from a representative Raw file (‘qualMedianDist’ function).

back to top

### 1.3.10 PG: Contaminant per condition

↓ Show Help

External protein contamination should be controlled for, therefore MaxQuant ships with a comprehensive, yet customizable protein contamination database, which is searched by MaxQuant by default. PTXQC generates a contamination plot derived from the proteinGroups (PG) table showing the fraction of total protein intensity attributable to contaminants. The plot employs transparency to discern differences in the group-wise summed protein abundance. This allows to delineate a high contamination in high complexity samples from a high contamination in low complexity samples (e.g. from in-gel digestion). If you see only one abundance class (‘mid’), this means all your groups have roughly the same summed protein intensity. Note that this plot is based on experimental groups, and therefore may not correspond 1:1 to Raw files.

Heatmap score: none (since data source proteinGroups.txt is not related 1:1 to Raw files)

back to top

### 1.3.11 MSMSscans: TopN

↓ Show Help

Reaching TopN on a regular basis indicates that all sections of the LC gradient deliver a sufficient number of peptides to keep the instrument busy. This metric somewhat summarizes ‘TopN over RT’.

Heatmap score [MS2 Scans: TopN high]: rewards if TopN was reached on a regular basis (function qualHighest)

back to top

### 1.3.12 MSMSscans: TopN over RT

↓ Show Help

TopN over retention time. Similar to ID over RT, this metric reflects the complexity of the sample at any point in time. Ideally complexity should be made roughly equal (constant) by choosing a proper (non-linear) LC gradient. See Moruz 2014, DOI: 10.1002/pmic.201400036 for details.

Heatmap score [MS2 Scans: TopN over RT]: Rewards uniform (function Uniform) TopN events over time.

back to top

### 1.3.13 EVD: IDs over RT

↓ Show Help

Judge column occupancy over retention time. Ideally, the LC gradient is chosen such that the number of identifications (here, after FDR filtering) is uniform over time, to ensure consistent instrument duty cycles. Sharp peaks and uneven distribution of identifications over time indicate potential for LC gradient optimization. See Moruz 2014, DOI: 10.1002/pmic.201400036 for details.

Heatmap score [EVD: ID rate over RT]: Scored using ‘Uniform’ scoring function, i.e. constant receives good score, extreme shapes are bad.

back to top

### 1.3.14 EVD: Peak width over RT

↓ Show Help

One parameter of optimal and reproducible chromatographic separation is the distribution of widths of peptide elution peaks, derived from the evidence table. Ideally, all Raw files show a similar distribution, e.g. to allow for equal conditions during dynamic precursor exclusion, RT alignment or peptide quantification.

Heatmap score [EVD: RT Peak Width]: Scored using BestKS function, i.e. the D statistic of a Kolmogoriv-Smirnoff test.

back to top

### 1.3.15 EVD: MBR - alignment

**alignment reference: 20200520\_QExHFX1\_RSLC1\_Anrather\_Hartl\_MFPL\_A1\_iST1\_iRT\_500ng**

↓ Show Help

MBR Alignment: First of two steps (1=align, 2=transfer) during Match-between-runs. This plot is based purely on real MS/MS ids. Ideally, RTs of identical peptides should be equal (i.e. very small residual RT delta) across Raw files after alignment.

MaxQuants RT correction is shown in blue – it should be well within the alignment search window (20min by default) set during MaxQuant configuration. The resulting residual RT delta after RT alignment (compared to a reference Raw file), is shown as green/red dots. One dot represents one peptide (incl. charge). Every dot (peptide) outside an allowed residual delta RT (1min by default) is colored red. All others are green.

If moving ‘red’ dots to the horizontal zero-line (to make them green) requires large RT shifts, then increasing the alignment search window might help MaxQuant to find a better alignment.

Heatmap score [EVD: MBR Align]: fraction of ‘green’ vs. ‘green+red’ peptides.

back to top

### 1.3.16 EVD: MBR - ID Transfer

↓ Show Help

MBR Transfer: Last of two steps (1=align, 2=transfer) during Match-between-runs. If MaxQuant only transfers peptide ID’s which are not present in the target file, then each Raw file should not have any duplicates of identical peptides (incl. charge). Sometimes, a single or split 3D-peak gets annotated multiple times, that’s ok. However, the same peptide should not be annotated twice (or more) at vastly different points in RT.

This plot shows three columns: - left: the ‘genuine’ situation (pretending that no MBR was computed) - middle: looking only at transferred IDs - right: combined picture (a mixture of left+middle, usually)

Each peptide falls into three categories (the colors): - single (good, because it has either one genuine OR a transferred ID). - in-group (also good, because all ID’s are very close in RT) - out-group (bad, spread across the RT gradient – should not be possible; a false ID)

Heatmap score [EVD: MBR ID-Transfer]: The fraction of non-out-group peptides (i.e. good peptides) in the middle column. This score is ‘pessimistic’ because if few ID’s were transferred, but all of them are bad, the score is bad, even though the majority of peptides is still ok (because they are genuine). However, in this case MBR provides few (and wrong) additional information, and should be disabled.

back to top

### 1.3.17 [experimental] EVD: Clustering Tree of Raw files

**by Correlation of Corrected Retention Times**

↓ Show Help

Auxililiary plots – experimental – without scores.

Return a tree plot with a possible alignment tree. This allows the user to judge which Raw files have similar corrected RT’s (i.e. where aligned successfully). If there are clear sub-clusters, it might be worth introducing artifical fractions into MaxQuant, to avoid ID-transfer between these clusters (use the MBR-Align and MBR-ID-Transfer metrics to support the decision).

If the input contains fractions, leaf nodes will be colored accordingly. Distinct sub-clusters should have their own color. If not, MaxQuant’s fraction settings should be optimized. Note that introducing fractions in MaxQuant will naturally lead to a clustering here (it’s somewhat circular).

Heatmap score: none.

back to top

### 1.3.18 MSMSscans: Ion Injection Time over RT

↓ Show Help

Ion injection time score - should be as low as possible to allow fast cycles. Correlated with peptide intensity. Note that this threshold needs customization depending on the instrument used (e.g., ITMS vs. FTMS).

Heatmap score [MS2 Scans: Ion Inj Time]: Linear score as fraction of MS/MS below the threshold.

back to top

### 1.3.19 [experimental] MSMSscans: MS/MS intensity

↓ Show Help

MS/MS identifications can be ‘bad’ for a couple of reasons. It could be computational, i.e. ID rates are low because you specified the wrong protein database or modifications (not our concern here). Another reason is low/missing signals for fragment ions, e.g. due to bad (quadrupole/optics) ion transmission (charging effects), too small isolation windows, etc.

Hence, we plot the TIC and base peak intensity of all MS/MS scans (incl. unidentified ones) per Raw file. Depending on the setup, these intensities can vary, but telling apart good from bad samples should never be a problem. If you only have bad samples, you need to know the intensity a good sample would reach.

To automatically score this, we found that the TIC should be 10-100x larger than the base peak, i.e. there should be many other ions which are roughly as high (a good fragmentation ladder). If there are only a few spurious peaks (bad MS/MS), the TIC is much lower. Thus, we score the ratio BP \* 10 > TIC (this would be 100% score). If it’s only BP \* 3 < TIC, we say this MS/MS failed (0%). Anything between 3x and 10x gets a score in between. The score for the Raw file is computed as the median score across all its MS/MS scans.

Heatmap score [MS2 Scans: Intensity]: Linear score (0-100%) between 3 < (TIC / BP) < 10.

back to top

### 1.3.20 EVD: Oversampling (MS/MS counts per 3D-peak)

↓ Show Help

An oversampled 3D-peak is defined as a peak whose peptide ion (same sequence and same charge state) was identified by at least two distinct MS2 spectra in the same Raw file. For high complexity samples, oversampling of individual 3D-peaks automatically leads to undersampling or even omission of other 3D-peaks, reducing the number of identified peptides. Oversampling occurs in low-complexity samples or long LC gradients, as well as undersized dynamic exclusion windows for data independent acquisitions.

Heatmap score [EVD: MS2 Oversampling]: The percentage of non-oversampled 3D-peaks.

back to top

### 1.3.21 EVD: Uncalibrated mass error

↓ Show Help

Mass accurary before calibration. Outliers are marked as such (‘out-of-search-tol’) using ID rate and standard deviation as additional information (if available). If any Raw file is flagged ‘failed’, increasing MaxQuant’s first-search tolerance (20ppm by default, here: 20.0 ppm) might help to enable successful recalibration. A bug in MaxQuant sometimes leads to excessively high ppm mass errors (>104) reported in the output data. However, this can sometimes be corrected for by re-computing the delta mass error from other data. If this is the case, a warning (‘bugfix applied’) will be shown.

Heatmap score [EVD: MS Cal Pre (20.0)]: the centeredness (function CenteredRef) of uncalibrated masses in relation to the search window size.

back to top

### 1.3.22 EVD: Calibrated mass error

↓ Show Help

Precursor mass accuracy after calibration. Failed samples from precalibration data are still marked here. Ppm errors should be centered on zero and their spread is expected to be significantly smaller than before calibration.

Heatmap score [EVD: MS Cal-Post]: The variance and centeredness around zero of the calibrated distribution (function GaussDev).

back to top

### 1.3.23 MSMS: Fragment mass errors per Raw file

↓ Show Help

MS/MS decalibration metric. If most of the fragments are within tighter bounds, you can reduce the fragment mass tolerance to obtain more identifications under the same FDR. On the other hand, if the fragment mass errors are not centered on zero, a recalibration of the instrument should be performed. If the (Gaussian-like) distribution is cut too severely on either side by the search tolerance window in MaxQuant, you might be able to increase the number of identifications by allowing for a wider MS/MS search window when re-running MaxQuant. However, the number of decoy identifications will increase as well, potentially offsetting any gain when FDR is applied.

Heatmap score [MSMS: MS2 Cal (Analyzer)]: rewards centeredness around 0 ppm/Da (function Centered).

back to top

### 1.3.24 SM: MS/MS identified per Raw file

↓ Show Help

MS/MS identification rate per Raw file from summary.txt (SM). Each Raw file is colored according to its ID rate and categorized into performance bins as ‘bad’, ‘ok’ and ‘great’. Raw files below ‘ok’, are listed separately on the next page of the report for convenient follow-up.

The thresholds for the bins are

- bad (<20%)
- ok (20-35%)
- great (>35%)

Heatmap score [SM: MS2 IDrate (>35)]: reaches 1 (=100%) if the threshold for ‘great’ is reached or exceeded.

back to top

### 1.3.25 MSMSscans: TopN % identified over N

↓ Show Help

Looking at the identification rates per scan event (i.e. the MS/MS scans after a survey scan) can give hints on how well scheduled precursor peaks could be fragmented and identified. If performance drops for the later MS/MS scans, then the LC peaks are probably not wide enough to deliver enough eluent or the intensity threshold to trigger the MS/MS event should be lowered (if LC peak is already over), or increased (if LC peak is still to weak to collect enough ions).

Heatmap score [MS2 Scans: TopN ID over N]: Rewards uniform identification performance across all scan events.

back to top

### 1.3.26 [experimental] EVD: Non-Missing Peptides

**compared to all peptides seen in experiment**

↓ Show Help

Missing peptide intensities per Raw file from evidence.txt. This metric shows the fraction of missing peptides compared to all peptides seen in the whole experiment. The more Raw files you have, the higher this fraction is going to be (because there is always going to be some exotic [low intensity?] peptide which gets [falsely] identified in only a single Raw file). A second plot shows how many peptides (Y-axis) are covered by at least X Raw files. A third plot shows the density of the observed (line) and the missing (filled area) data. To reconstruct the distribution of missing values, an imputation strategy is required, so the argument is somewhat circular here. If all Raw files are (technical) replicates, i.e. we can expect that missing peptides are indeed present and have an intensity similar to the peptides we do see, then the median is a good estimator. This method performs a global normalization across Raw files (so their observed intensitiy distributions have the same mean), before computing the imputed values. Afterwards, the distributions are de-normalized again (shifting them back to their) original locations – but this time with imputed peptides.

Peptides obtained via Match-between-run (MBR) are accounted for (i.e. are considered as present = non-missing). Thus, make sure that MBR is working as intended (see MBR metrics).

**Warning:** this metric is meaningless for fractionated data! **TODO:** compensate for lower scores in large studies (with many Raw files), since peptide FDR is accumulating!?

Heatmap score [EVD: Pep Missing]: Linear scale of the fraction of missing peptides.

### 1.3.27 [experimental] EVD: Non-missing by set

### 1.3.28 [experimental] EVD: Imputed Peptide Intensity Distribution of Missing Values

back to top

### 1.3.29 EVD: Peptide ID count

↓ Show Help

Number of unique (i.e. not counted twice) peptide sequences including modifications (after FDR) per Raw file. A configurable target threshold is indicated as dashed line.

If MBR was enabled, three categories (‘genuine (exclusive)’, ‘genuine + transferred’, ‘transferred (exclusive)’ are shown, so the user can judge the gain that MBR provides.

If MBR would be switched off, you can expect to see the number of peptides corresponding to ‘genuine (exclusive)’ + ‘genuine + transferred’. In general, if the MBR gain is low and the MBR scores are bad (see the two MBR-related metrics), MBR should be switched off for the Raw files which are affected (could be a few or all).

Heatmap score [EVD: Pep Count (>15000)]: Linear scoring from zero. Reaching or exceeding the target threshold gives a score of 100%.

back to top

### 1.3.30 EVD: ProteinGroups count

**MBR gain: +11%**

↓ Show Help

Number of Protein groups (after FDR) per Raw file. A configurable target threshold is indicated as dashed line.

If MBR was enabled, three categories (‘genuine (exclusive)’, ‘genuine + transferred’, ‘transferred (exclusive)’ are shown, so the user can judge the gain that MBR provides. Here, ‘transferred (exclusive)’ means that this protein group has peptide evidence which originates only from transferred peptide IDs. The quantification is (of course) always from the local Raw file. Proteins in the ‘genuine + transferred’ category have peptide evidence from within the Raw file by MS/MS, but at the same time also peptide IDs transferred to this Raw file using MBR were used. It is not unusual to see the ‘genuine + transferred’ category be the rather large, since a protein group usually has peptide evidence from both sources. To see of MBR worked, it is better to look at the two MBR-related metrics.

If MBR would be switched off, you can expect to see the number of protein groups corresponding to ‘genuine (exclusive)’ + ‘genuine + transferred’. In general, if the MBR gain is low and the MBR scores are bad (see the two MBR-related metrics), MBR should be switched off for the Raw files which are affected (could be a few or all).

Heatmap score [EVD: Prot Count (>3500)]: Linear scoring from zero. Reaching or exceeding the target threshold gives a score of 100%.

back to top

### 1.3.31 EVD: UpSet distinct

↓ Show Help

The metric shows an upSet plot based on the number of modified peptide sequences per Raw file, intersected or merged with other Raw files (see below for details).

If the number of Raw files is >=6, only the `distinct` plot is generated (the other two are skipped for performance reasons). See here for an example plot showing how the set size is computed .

Definition: An `active set` is the set of black dots in a column of the plot – as opposed to the grey dots (you will understand when you see it).

**distinct:** shows the number of sequences that are present in ALL active sets. For three Raw files and active sets A and B, this would mean all sequences which occur in A and B (intersect), but not in C (setdiff).  
 **intersection:** shows the number of sequences that occurs in all active sets (intersection).  
 **union:** shows the number of sequences that occurs in total. For two files that are all sequences that occurs either in A or in B (union).  

Heatmap score [EVD: UpSet]: The proportion of sequences that the file has in common with all other files.

back to top
