# Supplementary figures and images for: In Search of a Universal Method: A Comparative Survey of Bottom-Up Proteomics Sample Preparation Methods
Source: J Proteome Res. 2022 Aug 25;21(10):2397–411. doi: 10.1021/acs.jproteome.2c00265 (PMC9552232; doi:10.1021/acs.jproteome.2c00265)

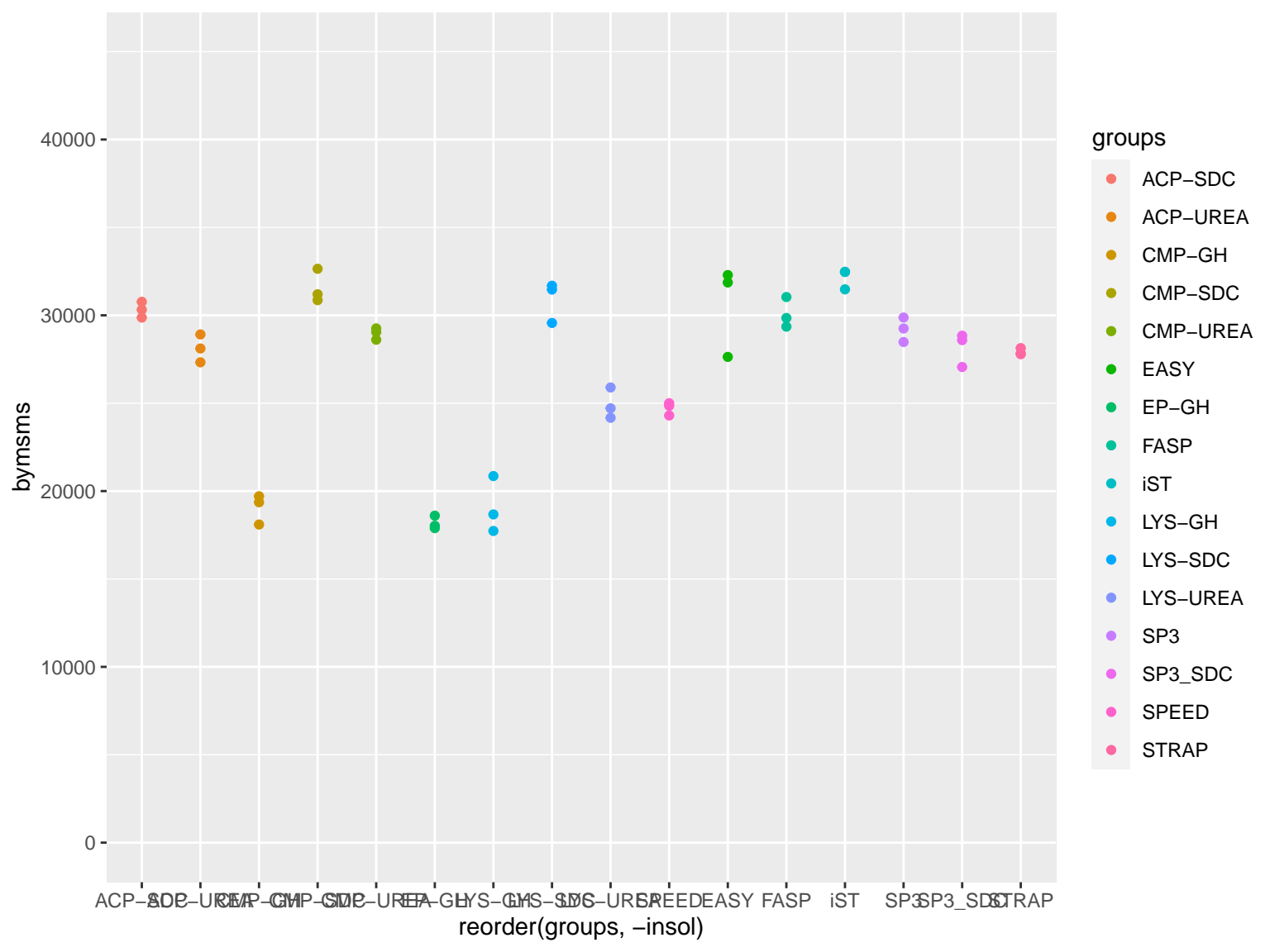

Supplement: Supplementary file 5 — pr2c00265_si_005.zip [file pr2c00265_si_005.zip › Supplemental_Material_Scripts/Batch_Bridging/corrected.pdf]

## nr IDs of bridge measurements

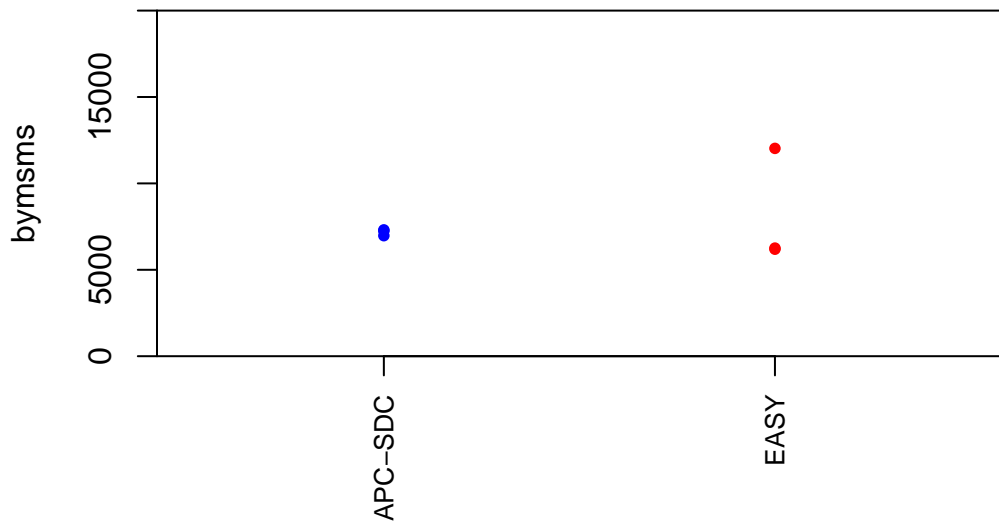

Supplement: Supplementary file 5 — pr2c00265_si_005.zip [file pr2c00265_si_005.zip › Supplemental_Material_Scripts/Batch_Bridging/overview.pdf]
